# Gallic acid based green corrosion inhibitor for mild steel in 1 M HCl electrochemical and microbial assessment with theoretical validation

**DOI:** 10.1038/s41598-025-97647-3

**Published:** 2025-04-30

**Authors:** Ahmed. E. Suliman, Ahmed H. Mangood, Naema S. Yehia, M. Abdelraouf, Hany A. Batakoushy, Moaz M. Abdou, E. G. Zaki

**Affiliations:** 1 Burg Al-arab Petroleum Company (Burapetco) 204 A ST 287, New Maadi, Cairo, Egypt; 2 Chemistry Department, Faculty of Science, Menoufia University, Shibin El Kom, Menouia Egypt; 3 Egyptian Petroleum Research Institute, Nasr City, Cairo 11727 Egypt; 4 Center for Scientific Research and Sustainable Development, Sinai University, Kantra Branch, Ismailia, Egypt; 5 Department of Pharmaceutical Analytical Chemistry, Faculty of Pharmacy, Menoufia University, Shibin El Kom, 32511 Egypt; 6 Faculty of Postgraduate Studies for Nanotechnology, Cairo University, El-Sheikh Zayed, Giza, 12588 Egypt; 7 Department of Pharmaceutical Analytical Chemistry, Faculty of Pharmacy, Menoufia National University, 70th km Cairo-Alexandria Agricultural Road, Menoufia, Egypt

**Keywords:** Gallic acid, Corrosion, Tafel polarization, EIS, Dynamic simulation, Molecular docking, Environmental sciences, Materials science

## Abstract

The petroleum industry, characterized by the significant investment in costly equipment and devices utilized in the extraction, production, or processing of crude oil, can result in the loss of valuable assets or the crude itself. This research involved the synthesis of a Schiff base from substituted gallic acid derivatives through an intermediate reaction known as N-(2-{2-[2-(2-amino-ethylamino)-ethylamino]-ethylamino}-ethyl)-3,4,5-trihydroxy-benzamide (AEET). The synthesized compound was characterized using FTIR and 1HNMR spectroscopy to evaluate its effectiveness in inhibition. The performance of the inhibitors was assessed through an electrochemical process that included Tafel and EIS. This evaluation was supported by theoretical mechanisms involving density functional theory (DFT) and molecular dynamics simulations (MDS). To validate the findings from the electrochemical studies, the scanning electron microscopy (SEM) technique was employed to examine the topographic anisotropy characteristics between the treated and untreated samples of mild steel species. The bioassay diluted serial technique was utilized to assess the AEET as effective biocides for managing bacterial growth issues. This evaluation included an analysis of the AEET’s efficiency in inhibiting sulfate-reducing bacteria (SRB). Additionally, computational methods were described, demonstrating optimal scores, RMSD values, and binding interaction energies associated with the formation of hydrogen bonds with specific receptor residues to investigate the biological activity.

## Introduction

The scientific study of corrosion inhibitors has increased from being a selective issue to becoming a topic of considerable discussion and research with the goal of discovering effective techniques to reduce the danger of recurrence. Mild steel is utilized as a construction material in a variety of industrial sectors, particularly the petroleum industry, due to the valuable mechanical features that it possesses^1,2^. When it comes to pipe operation, internal corrosion is typically a considerable be concerned, and the primary cause of this concern is stress corrosion cracking^3^. There has been a recent increase in galvanic corrosion issues that have been linked to the utilization of materials that are not equivalent, which has garnered a substantial amount of attention^4^. Also, there are the typical issues that are associated with wells, such as failures in the materials, but there are also corrosion issues that develop in the annular region that is located between the steel tubing and the casing, which becomes clogged. Cracks caused by corrosion can develop as a result of these attacks over time^5,6^. Numerous scientific investigations have been carried out by researchers in order to address the impacts of metal corrosion, and they have also introduced novel compounds in order to improve the resistance of materials^5–8^. When compared to other methods of mitigating corrosion, such as coating and plastic deformations, corrosion inhibitors are the most appropriate strategy for protecting metal surfaces by enhancing their capacity to withstand the corrosion process^7^. : The ability of these compounds to adhere to the surface of mild steel is facilitated by the presence of a multitude of electron-donating atoms, including S, N, O, and P, as well as π-bonds or aromatic rings^9–12^. As a result of the fact that these electrons like to interact with metal surfaces in acidic conditions, the incorporation of these atoms into heterocyclic compounds results in considerable enhancement of the corrosion process. In order to enhance the efficiency of inhibition, it is possible to incorporate heterocyclic atoms and π-bonds into intermolecular structures^7,13^. Bonds are formed as a result of the adsorption process, which can be either chemical or physical, depending on whether the organic inhibitor and the metal surface are strongly attracted to one another^14,15^. When compared to its building structures, such as aldehyde and amine compounds, Schiff bases have been discovered by researchers to have significantly higher inhibitory efficiency^16,17^. The low toxicity and inexpensive cost of these chemicals are the reasons for their widespread application^18,19^. In order to establish whether or not a compound is helpful in reducing corrosion rates, the primary factor that is considered is whether or not it can adsorb onto the surface of the metal^20,21^. The selection of an efficient anti-corrosive agent is largely influenced by the research findings obtained from experiments. For the purpose of optimizing the protective qualities of the chemical in real-world applications, this selection procedure frequently involves the analysis of a variety of criteria, such as the molecular structure, concentration, and environment^22^. At the moment, we are utilizing theoretical analyses to evaluate the accessibility of experimental data by contrasting measurements that are empirical and those that are quantum^23^. Therefore, the purpose of this work was to develop a new Schiff base compound with the intention of investigating the inhibitory process that it faced in the process of corrosion. Computational simulation, which can look into corrosion inhibitor molecules using DFT, MD simulation, and molecular docking methods in both their liquid and gas phase conditions, is one of the many tools that can be used to study molecular structures that come into contact with a solution that contains 1 M of HCl and an MS surface. Other possible tools include molecular docking, molecular dynamics, and nuclear magnetic resonance. These findings provide evidence that there is a correlation between the modeling of prepared molecular structures and the capacity of such structures to prevent corrosion.

## Experimental

### Synthesis of AEET inhibitor

**Scheme 1:**
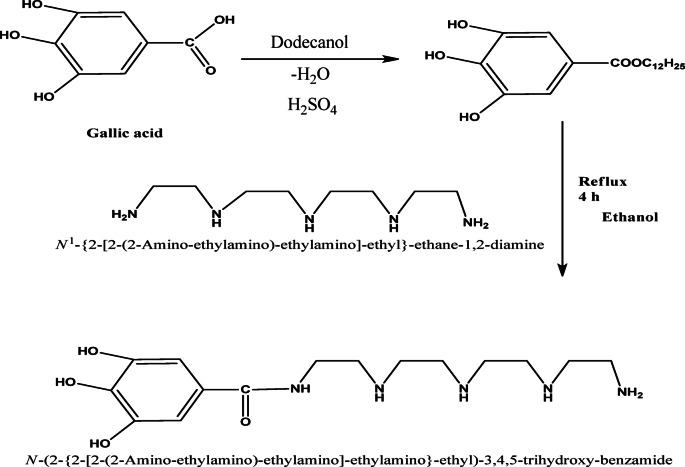
Procedure to prepare AEET compound.

The studied inhibitor was synthesized via a reaction between the gallic acid and dodecanol to obtain dodecyl gallate after adding two drops of H₂SO₄, then substituted the ester of gallic acid with a concentration of 97% that reacts with diamines (N1-{2-[2-(2-amino-ethylamino)-ethylamino]-ethyl}-ethane-1,2-diamine) with a concentration of 99% to form a solution with a 1:1 M ratio. The compound is named N-(2-{2-[2-(2-amino-ethylamino)-ethylamino]-ethylamino}-ethyl)-3,4,5-trihydroxy-benzamide (AEET). The preparation process of the compound is shown in Scheme 1.

The chemical structure was confirmed by, IR spectra (KBr discs) were recorded on Thermo Scientific Nicolet iS10 FTIR (BRUKER) spectrometer. The 1 H NMR (500 MHz) spectra was recorded on a JEOL spectrometer (ECZR version) using DMSO-d6 as solvents.

### Materials and methods

Cylindrical (MS) specimens with the following chemical composition were used to perform the corrosion measurements: wt% (P, Si, Mn, C, S) elements represent 0.019%, 0.246%, 0.47%, 0.167%, and 0.018%, respectively, and the remaining Fe. The cylindrical (MS) were polished using fine-grain emery paper (grade 400 to 2000), followed by a wash in distilled water and drying at room temperature very well. Solution represents concentration. 37% of HCl was diluted to be 1 M of HCl by distilled water. To confirm the solubility of the AEET compound was achieved, a ratio of solution containing 20:80 of ethanol. The stock solution was prepared with 1 M HCl, and different concentrations (7 × 10^− 5^- 5 × 10^− 4^ M) were obtained. The Gallic acid purchased from Sigma-Aldrich Company, diamines (N1-{2-[2-(2-amino-ethylamino)-ethylamino]-ethyl}-ethane-1,2-diamine) purchased from Merck Company.

### Corrosion measurements

#### Electrochemical measurements

The Origamaster 5.0 potentiostat/galvanostat was implemented to perform the experiment of electrochemical measurements. Three electrodes are used in the system contained in a Pyrex glass cell: the (MS) electrode with an exposed surface area of 0.384 cm² used as the working electrode, Pt (platinum) acting as an auxiliary electrode, and Ag/AgCl acting as a working electrode, respectively. It was implemented to study the electrochemical measurements of (MS) without the attendance of the prepared (AEET) Schiff bases compound at 298 K to the working electrode, which was ready-fitted by the Origalys (2.4.0.7) software. Open circuit potential (OCP) appeared after 30 min, and electrochemical impedance spectroscopy (EIS) measurements frequency ranged from 100 kHz to 0.1 Hz. The Tafel measurement’s potential range is ± 300 mV with a scan rate of 2 mV/s.

### SEM observation

The scanning electron microscope model ZEISS - EVO 15 – UK device was implemented to observe the morphology of the (MS) surface that was treated with a concentration (5 × 10^− 4^ M) of the AEET compound, also without treatment for 6 h. The images of metal sheets appear to show the surface smooth and rough morphology with the study of the SEM observation.

### Computational studies

BIOVIA Materials Studio (20.1) software. was implemented to carry out the computational studies. The obtained geometry optimization simulation of (AEET) was performed by using the DMol3 tool with suitable cutoffs. The optimal inhibitor structures were conducted by using the DFT computation electronic structures to become ready to extract the RDFs analysis, interaction, and binding energies by contacted (MD) simulation.

### Biocidal measurements against sulfate-reducing bacteria (SRB)

The inhibition of SRB microorganism growth in anaerobic environments was assessed utilizing the serial dilution technique outlined in ASTM D4412-84. We collected raw samples of water infected by SRB from the concession depths of the Burg AL Arab Petroleum Co. oil production tank in the West Desert of Egypt. This anaerobic water provides an ideal habitat for SRB colonization. In such an environment, SRB can cause significant damage through microbial-induced corrosion (MIC), potentially leading to catastrophic consequences. To combat SRB, we examined the efficacy of a biocide called AEET at two different concentrations: 1 × 10^− 4^ M and 2 × 10^− 4^ M. The experiment was conducted over 21 days at 40 °C. The biocidal activity of AEET was evaluated following the standardized test method NACE TM0194-14-SG.

### Molecular docking studies

ChemDraw 2014 was used to sketch the tested compounds, and docking studies were implemented to describe the biological activity results of AEET tested compounds based on the interaction of ligand–protein receptors using MOE software. Docking analysis was performed on the active pocket sites of different crystal protein structures obtained using the Protein Data Bank website to match the experimental results conducted here to overcome the SRB issues. Docking studies were carried out using triangle matcher, and the refinement methodology is based on rigid receptors, and the scoring methodology is GBVI/WSA dG to select the best poses and the best acceptable RMSD values.

## Result and discussion

### Structure characterization

**Fig. 1:**
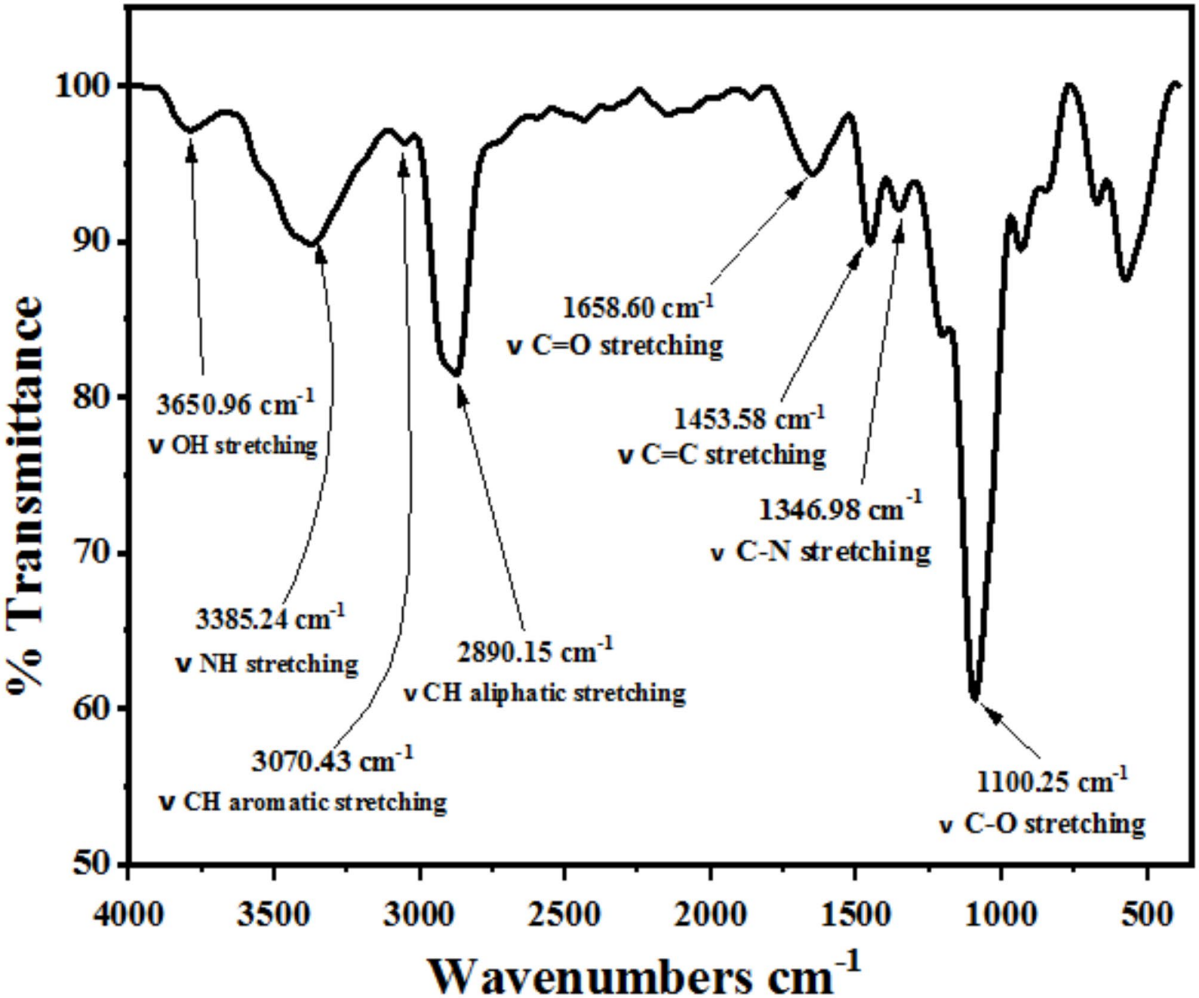
FTIR spectrum of AEET compound.

FTIR: the chemical structure of AEET was validated through the using Fourier transform infrared measurements with an exposed region as shown in Fig. 1. Exhibited band at 3650.96 cm^− 1^(ν OH stretching), 3385.24 cm^− 1^(ν NH stretching), 3070.43 cm^− 1^(*ν* CH-aromatic stretching), 2890.15 cm^− 1^(*ν* CH-aliphatic stretching), 1658.60 cm^− 1^(*ν* C = O stretching), 1453.85 cm^− 1^(*ν* C = C aromatic), 1346.98 cm^− 1^(ν C-N stretching) and 1100.25 cm^− 1^(ν C-O phenolic oxygen stretching).

**Fig. 2:**
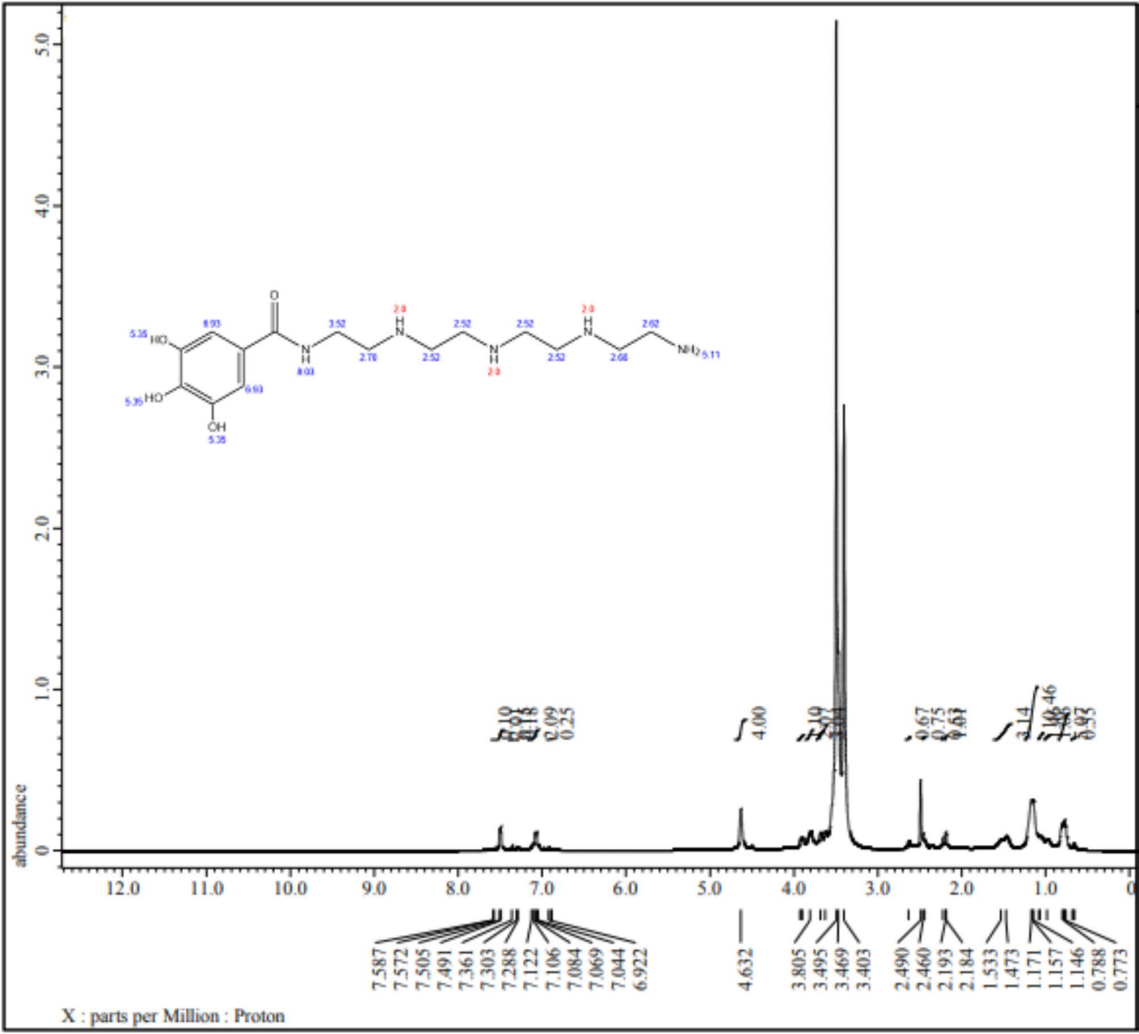
^1^HNMR spectrum of AEET compound.

^1^H NMR (500 MHz, DMSO-d6) spectrum of AEET was characterized as Fig. 2. δ (ppm): 1.47–1.50 (s, 3 H, NH), 2.18, 2.19,2.24 (s, 2 H,3CH2),2.63(s, 2 H, CH2), 3.80 (br.,2 H, NH2), 3.68 (s, 2 H, CH2), 4.46 (s, 2 H, CH2),4.59–4.61 (s, 4 H, 2CH2), 7.06–7.08 (d,2 H, Ar-H), 7.27 (s,1H, NHCO), 7.47–7.49 (d,2 H, Ar-H) Analy.Calcud. for Formula C15H27N5O4 (341.41).

### Potentiodynamic polarization (PDP) measurements

**Fig. 3:**
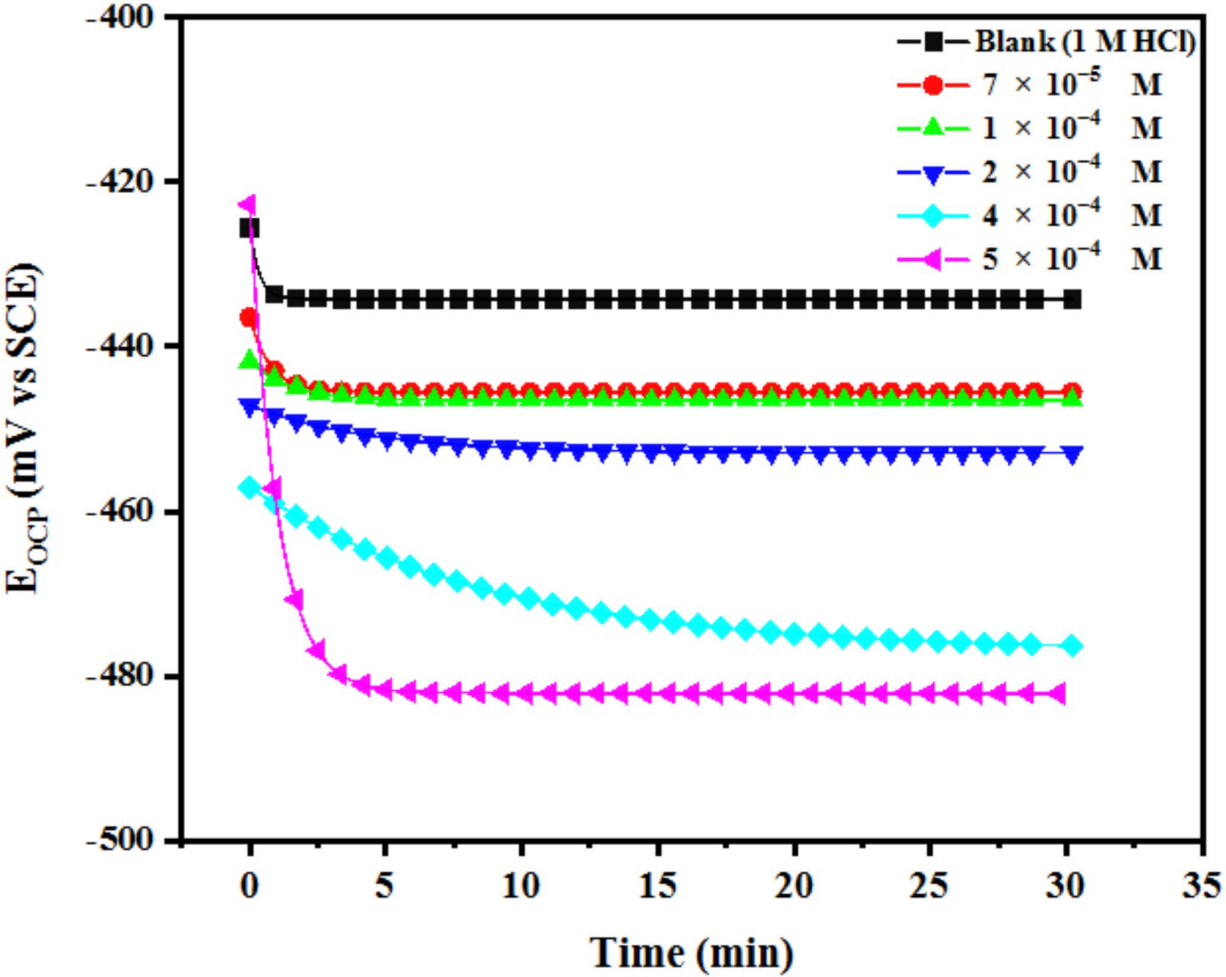
The OCP- Time curves for the (MS) electrode at various concentrations of AEET in 1 M HCl at room temperature.

After performing OCP measurements and reaching a steady state of potential, the potentiodynamic polarization method was used to examine the corrosion resistance of AEET in 1 M HCl as a blank and with inhibition solutions on the MS surface, as shown in Fig. 3^24,25^. The OCP measurements revealed minimal variation between the blank and inhibited samples, with fluctuations not exceeding ± 85 mV. This indicates the stability of the electrochemical system and supports the reliability of the E_corr_ values presented in Table 1.

The lower current density (i) was observed to shift towards both the cathodic and anodic sides, as demonstrated by the polarization curves, with no significant alteration in the potential (E_corr_). This method illustrates how the generated inhibitor interacts with the metal surface, reducing the interactions between the cathodic and anodic electrodes, functioning as mixed-type inhibitors^26,27^. The Tafel curve extrapolation method was employed to obtain various significant electrochemical parameters, such as potential corrosion (E_corr_), current density corrosion (Icorr), cathodic (β_c_) and anodic (β_a_) Tafel slopes, and corrosion efficiency (IE), all of which were determined from I_corr_ utilizing equation 0.1^28^. Table 1 presents the parameters.

1 $$\:EF=\frac{1-{\:\:\:\:\:I}_{corr}^{0}}{{I}_{corr}}$$

where $\:{I}_{corr}^{0}$ parameter introduce the current density values if found AEET inhibitor and absence, respectively.

**Fig. 4:**
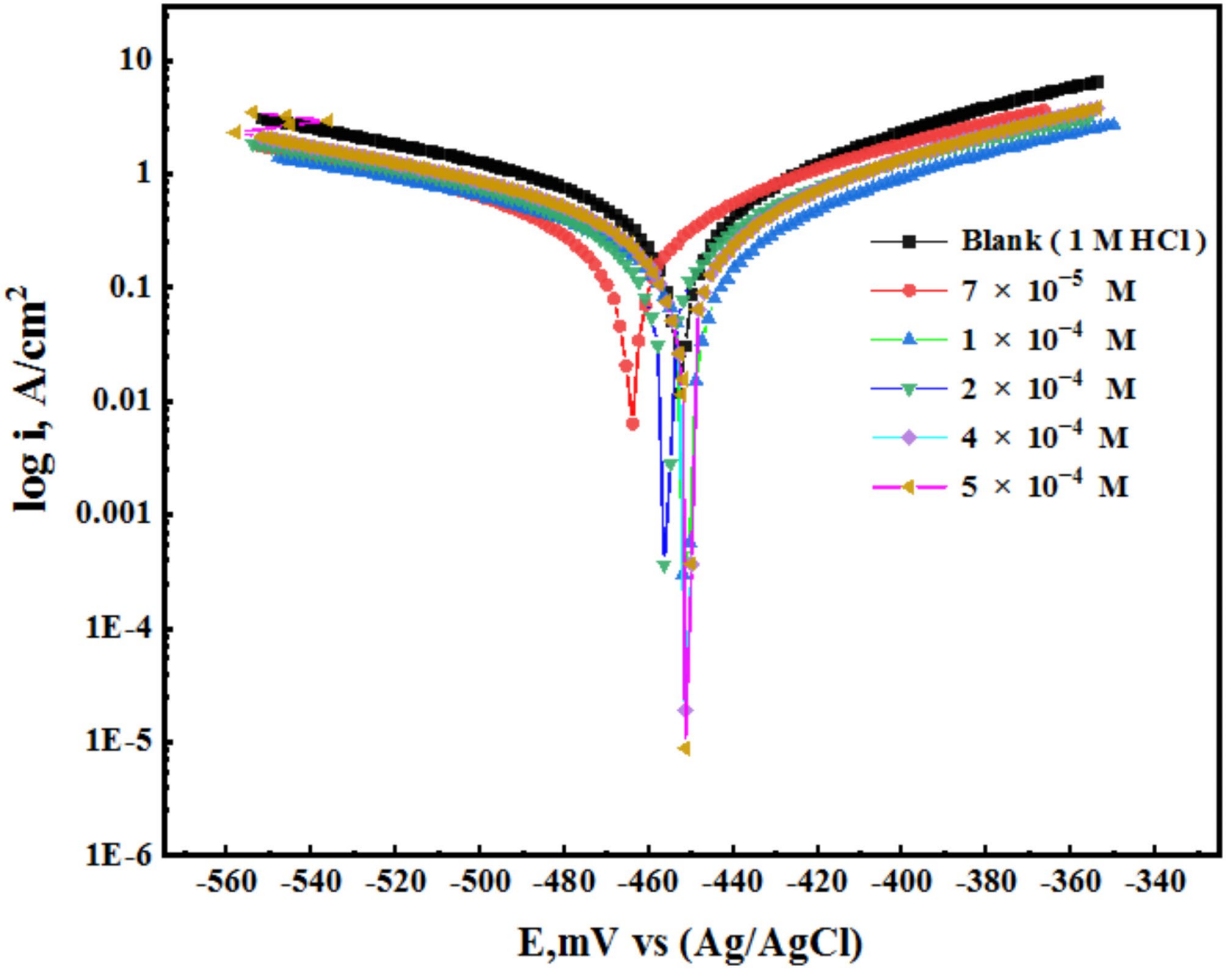
Polarization curves for the surface of (MS) in 1 M HCl with and without the different concentrations of (AEET) at room temperature .

The corrosion (CR) and corrosion current density (I_corr_) exhibited a decrease with an increase in the concentration of the AEET inhibitor. This observation indicates that the inhibitor effectively reduces the dissolution of the anodic metal surface. At the same time, the cathodic hydrogen atoms contribute to this process by segregating the surface area that is exposed to the electrolyte^29^. The E_corr_ values for both treated and untreated solutions exhibited a variation of less than 85 mV. As a result, no significant shift was observed, hence confirming the inhibition process^30^. Figure 4 illustrates that the polarization data obtained indicates a mixed type of corrosion behavior. The calculation of polarization resistance (R_p_) is performed using mathematical Eq. 2.

2 $$\:Rp\:=\frac{\beta\:a\:\times\:\:\beta\:c}{2.303\:{i}_{corr}(\beta\:a\:+\:\beta\:c)}$$

**Table 1:** The polarization parameters of the MS in the different concentrations of the AEET in 1 M HCl and the Inhibition efficiency.

Inh.	Conc.(M)	−E_corr_ (mV)	i_corr_ (µA.cm^− 2^)	−βc (mV.dec^− 1^)	βa (mV.dec^− 1^)	Rp (Ω. cm)	CR _(mpy)_	θ	IE_pot_ (%)
AEET	Blank	452.8	0.6162	138.5	89.1	176.02	6.2	–	–
	7 × 10^− 5^	469.1	0.3199	133.9	97.3	483.17	5.09	0.4808	48.08
	1 × 10^− 4^	462.9	0.2532	146.9	96.9	488.22	4.35	0.6783	67.83
	2 × 10^− 4^	462.2	0.1701	159.2	101.8	720.74	2.86	0.7583	72.39
	4 × 10^− 4^	452.5	0.0958	141.6	110.5	2280.37	1.58	0.8221	84.45
	5 × 10^− 4^	463.1	0.0613	155.6	109.2	2593.93	0.62	0.9389	90.05

### Electrochemical impedance spectroscopy

**Fig. 5:**
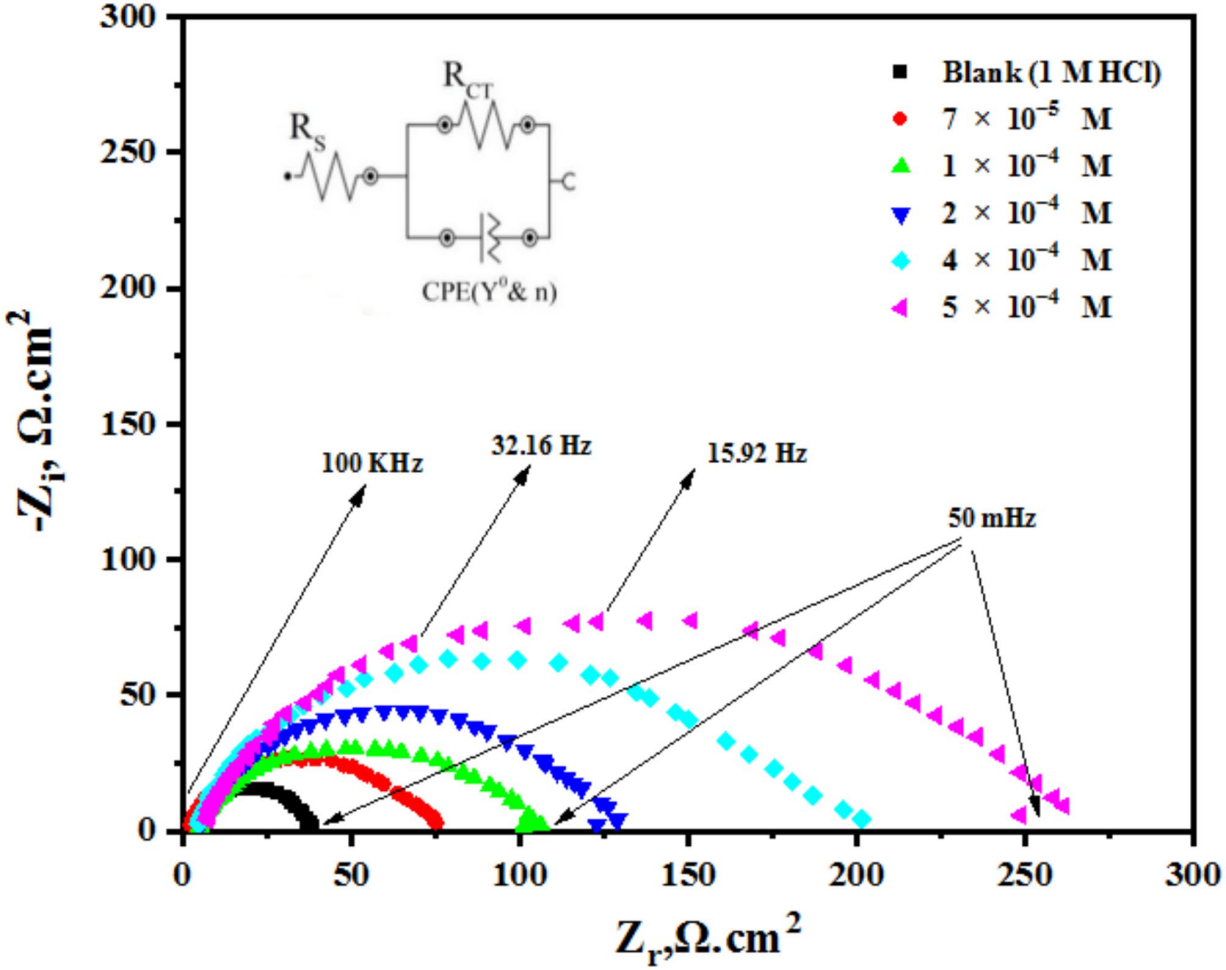
Nyquist plots for MS surfce in 1 M HCl in the absence and presence of various concentrations of AEET at room temperature.

**Fig. 6:**
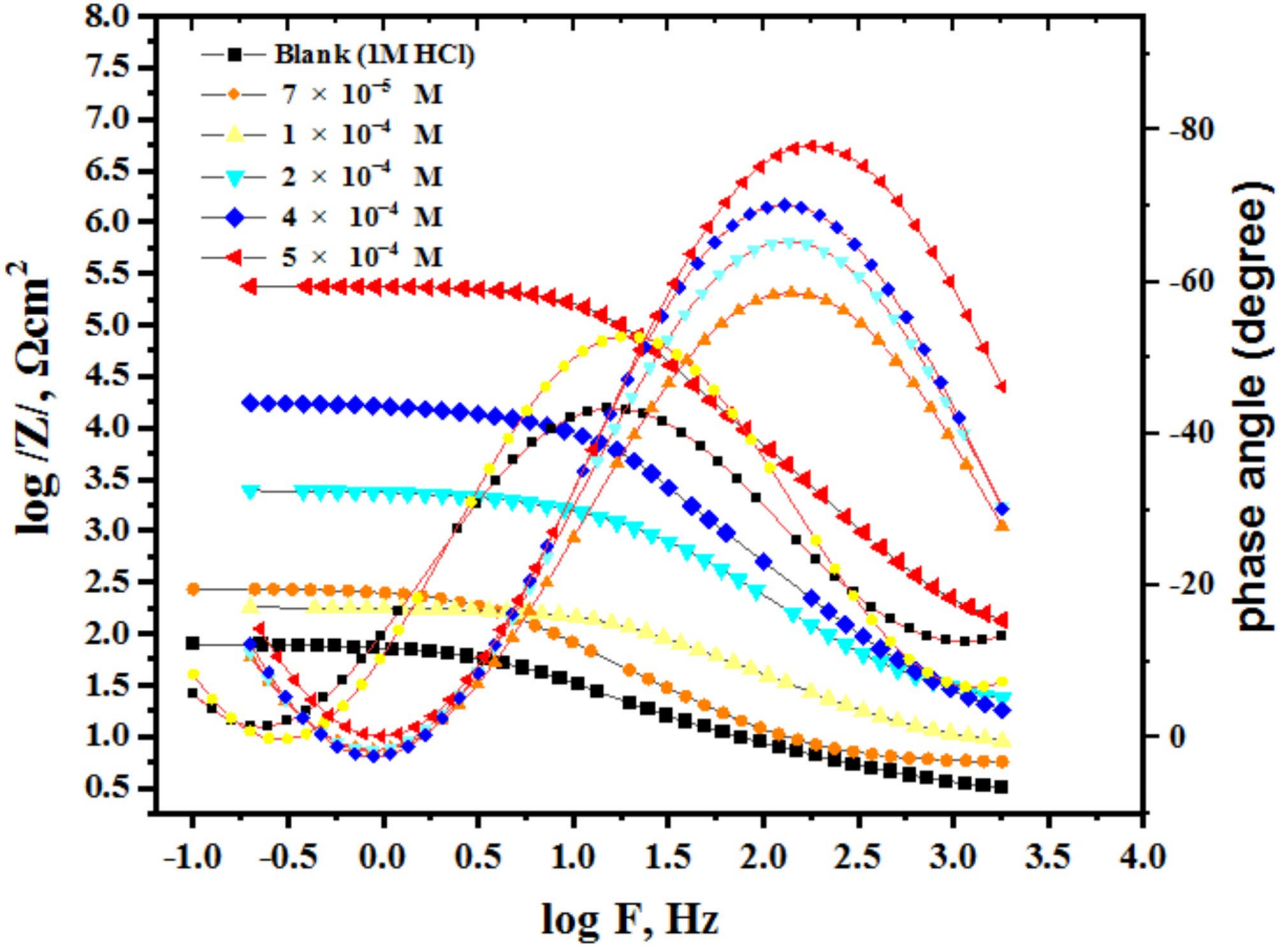
Bode and phase diagram for MS surface in 1 M HCl in the absence and presence of various concentrations of AEET at room temprature.

Electrochemical impedance spectroscopy (EIS) measurements are utilized to confirm the previously evaluated electrochemical behavior and to assess the capacitive properties at the mild steel (MS)/solution interface. Figure 5 present the Nyquist and Bode plots for the MS sample immersed in 1 M HCl, both without and with different concentrations of AEET compond. The Nyquist plots obtained demonstrate a consistent pattern, suggesting that the addition of AEET to the corrosive environment effectively mitigates MS corrosion while keeping its fundamental mechanism^31^. The Nyquist plots exhibit single semicircles at low frequencies, indicating that the corrosion reaction is primarily influenced by charge transfer resistance^31^. The increased exponent values (n) in Table 2 for the inhibited sample relative to the blank solution indicate that the inhibitor improves surface uniformity via adsorption. Figure 6 present the Bode plots (log Z vs. log F) and phase angle diagrams for MS in 1 M HCl, both with and without different concentrations of AEET^32^. The figures indicate that the absolute impedance |Z| in the low-frequency domain shows a significant increase, which confirms the enhanced inhibition efficiency at increased concentrations^33^. The enhancement results from the adsorption of AEET molecules on the MS surface, which effectively obstructs its active sites. The negative shift in phase angle values further corroborates the conclusion that these compounds primarily operate by creating a protective layer on the MS surface^34^. The double-layer capacitance (C_dl_) and inhibition efficiency (η%) are calculated using the following equations:.

3 $$\:\text{ƞ}\text{\%}=\frac{{(R}_{ct\left(inh\right)}^{0}-{R}_{ct})}{{R}_{ct\left(inh\right)}}\:\mathsf{x}\mathsf{\:}100$$

The resistances of the charge transfer for the treated and untreated experimental ($\:{R}_{ct}^{0}$) and (R_ct_) are determined by their respective values. Besides these parameters, the equivalent circuit contained a constant phase element (CPE), which is related to the electrochemical double-layer capacitance (C_dl_). The observed value of impedance related to the (CPE) was determined by calculating the below Eqs^35,36^.

4 $$\:{Z}_{CPE}=[\frac{1}{{\text{Y}}_{0}{\left(j\omega\:\right)}^{n}}]$$

where (Z_CPE_) is related to the impedance parameter of (CPE), (Y_0_) is the phase element constant of (CPE), (ω) is the parameter referring to angular frequency, the parameter (j) is related to imaginary number value, and (n) refers to the phase change; if the changed value represents (0 < *n* < 1), when *n* = 1, that refers to a genuine capacitor, and at *n* = 0, this means pure resistance impact. The CPE is included in the system’s double-layer capacitance, which is given by Eq. 5.

5 $$\:{\text{C}}_{dl}={\left({Y}_{0}\:{R}_{ct}^{1-n}\right)}^{1ln}$$

The parameters obtained from the impedance information as a nonlinear square are represented in Table 2. The represented data can observe the change of the R_ct_ values by increasing the concentration of AEET inhibitor, which can reach 90%. The best inhibition value for the AEET compound was achieved at the maximum concentration selected (5 × 10^− 4^M), which was the formation of a protective layer at the interface of the metal surface and solution^37^. In addition, the inhibition concentration could have completely reduced the double-layer capacitance. The main cause of this is the presence of inhibitor molecules in the solution, which replace the highest dielectric particles with the lowest ones in water-inhibitor molecules, which expand in thickness layer after time^38^. As a result, it would decrease the electron transfer number due to the prevention of the electrical double layer to form, which would cause a decrease in (CR) values. For the calculation of double-layer capacitance, these changes can be taken into account by a Helmholtz model Eq. 6^39^.

6 $$\:{\text{C}}_{dl}=\frac{{\epsilon\:}_{0}{\epsilon\:}_{s\:A}}{d}$$

Where (A) is the cathode’s surface area, (d) is the double layer’s thickness, (ε_s_) is the medium’s dielectric constant, and (ε_0_) is the air permittivity of the electric double layer, and (n) the value is the phase change was located within the range between 0.86 and 0.91, which also protects the metal surface from corrosion due to the exchange charges accumulating between the surface of the metal and the electrolyte^40^.

**Table 2:** EIS parameters and Inhibition efficiency values for MS surface in 1 M HCl with and without various concentrations of AEET compound at room temperature.

Inh.	Conc. (M)	*R*_s_ (Ω. cm^2^)	*R*_ct_ (Ω.cm^2^)	C_dl_ (F.cm^− 2^)	*N*	Q	X^2^	θ	IE_imp_ (%)
AEET	Blank	2.26	36	92.21	0.91	175.22	0.00328	–	**-------**
	7 × 10^− 5^	3.46	84	82.45	0.88	153.68	0.00387	0.5722	57.22
	1 × 10^− 4^	2.92	100	74.97	0.87	147.17	0.00459	0.6443	64.43
	2 × 10^− 4^	4.45	130	62.12	0.85	127.98	0.00648	0.7261	72.61
	4 × 10^− 4^	3.94	190	34.55	0.87	67.54	0.00564	0.8194	81.94
	5 × 10^− 4^	4.86	349	23.25	0.86	39.84	0.00585	0.8976	89.76

### Surface analysis (SEM)

**Fig. 7:**
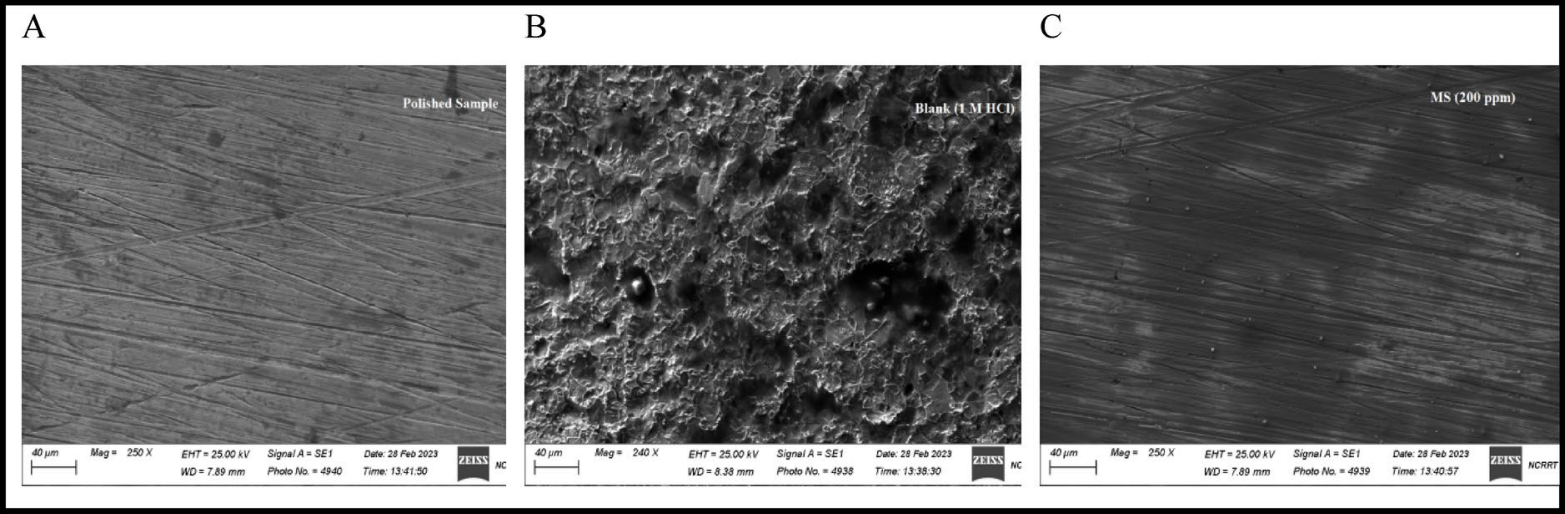
SEM image for MS surface: (**A**) finely polished sample, (**B**) sample immersed in 1 M HCl without inhibitor, (**C**) sample immersed in 1 M HCl with 5 × 10^− 4^M of AEET inhibitor.

The scanning electron microscope (SEM) was implemented to examine the morphology of the (MS) surface in a concentration of 1 M HCl solution in both treated and untreated conditions of the optimum concentration of the AEET compound, as shown in Fig. 7. The micrograph reveals that the surface is pitted and rough (cavity) when there is no inhibitor present because of immersion in the corrosive fluid (1 M HCl), which causes the corrosion process to take place^41,42^. Conversely, the AEET inhibitor effectively reduces the corrosion rate, as evidenced by the decreased presence of localized corrosion sites and the formation of a protective thin film that extensively covers the alloy surface. This material also did not show any signs of a pitted surface, perhaps because a smooth film formed on the surface and may be referred to as a protective layer^43,44^. Based on SEM analysis, the presence of the investigated inhibitors effectively prevents corrosion on the electrode surface by forming an adsorbed protective layer^45,46^. The inhibition efficiency results derived from both chemical and electrochemical methods further confirm the protective nature of this film in mitigating rust formation.

### Computational analysis

#### Quantum chemical calculations

**Fig. 8:**
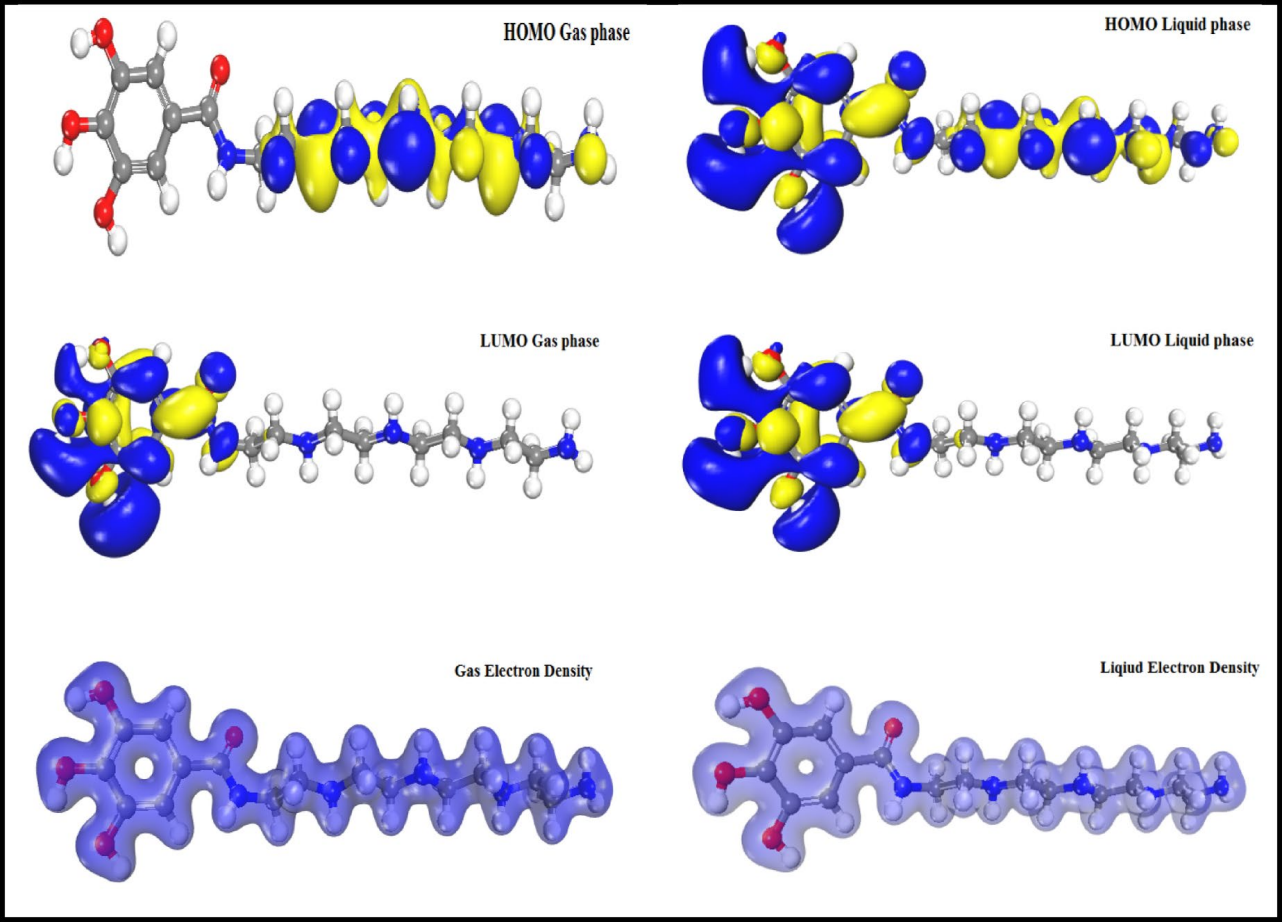
The molecular orbitals outputs for AEET compound in HOMO (upper), LUMO (middle), and total electron distributions (lower).

The electronic properties of the inhibitor are pivotal in defining its performance and in estimating the adsorption and deformation behavior of compounds on metal surfaces^10^. This paper aims to evaluate the relationship between computational studies, including molecular structures and experimental tools via DFT, in assessing the physical and chemical properties of Schiff base compounds^47^. The capacity of a compound to either lose or accept electrons is associated with frontier molecular orbitals (FMOs), specifically the Highest Occupied Molecular Orbital (HOMO) and the Lowest Unoccupied Molecular Orbital (LUMO)^48^. Figure 8 illustrates the electron density distribution of the HOMO and LUMO in the AEET compound within the gas phase, positioned in the substituted Schiff base chain and connected to the phenyl ring. In the case of LUMO, the electron density is positioned across the phenyl ring of the molecule. The hydroxyl groups in the AEET compound exhibit a high capacity for chemical interaction, attributed to an increase in the electronic cloud that facilitates electronic donation. The efficiency of corrosion inhibitor compounds is depending upon an electrostatic mechanism that involves the donation and acceptance of electrons during a chemical reaction. The capacity to accept electrons can be characterized by the distribution of the highest occupied molecular orbital (HOMO) in the liquid phase. The HOMOs are uniformly distributed throughout the molecule, while the LUMOs are localized within the phenyl ring. The molecular structure measurements of HOMO and LUMO indicate that the AEET compound exhibits significant responses to ion charge transfer substitution. Consequently, the AEET compound shows promise as a corrosion inhibitor molecule. Based on previous analysis, the energy values can be calculated, with (I) expressing the ionization energy and (E) denoting electron attraction, as determined by the following equations^49^:

7 $$\:E=\:{-E}_{\left(LUMO\right)}$$

8 $$\:I=\:-{E}_{\left(HOMO\right)}$$

We can represent the (I) and (E) values through using parameters to be useful in determining the findings, such as the reaction chemical potential (µ), absolute electronegativity value (x), and absolute hardness element (η), as in the following equations^49^.

9 $$\:\chi\:=-\:\mu\:=\frac{I+E}{2}$$

10 $$\upeta=\frac{\text{I}-\text{E}}{2}$$

11 $$\:\sigma\:\:=\frac{1}{{\upeta}}$$

The computed quantum parameters of the investigated (AEET) molecule in their gas and liquid form are represented in Table 3. According to the above-mentioned, HOMO refers to electron donating, and thus, a high E_HOMO_ value indicates an increased probability of donating electrons. On the other hand, LUMO refers to electron accepting, and it is widely known that the lower value of E_LUMO_ refers to a high acceptance of electron capacity^50,51^. From Table 3, the electron-donating ability matches with our experimental data conducted. The HOMO-LUMO gap (∆E) is one important indicator that determines the stability of the compound^52,53^. Regarding our results, it can be considered that the calculated gap energy indicates the probability of the AEET compound to act as a corrosion inhibitor to the protection surface of (MS) against corrosion attack. The observed measurements that indicate the corrosion inhibitor’s efficiency can be discussed based on the energy gap, which is essential from a theoretical point of view^53,54^. Therefore, based on the results we obtained, we can summarize that the presence of three hydroxyl and amino groups located in the AEET molecule has a greater effect on the liquid phase efficiency than the gas phase one. The following equations were used to determine the electrophilic value (ω) and the nucleophilic value (ε)^49^:

12 $$\:\omega\:=\frac{{\mu\:}^{2}}{2Ƞ}$$

13 $$\:\epsilon\:\:=\frac{1}{\omega\:}$$

The ability of molecules to accept electrons can be explained by using their electrophilicity as well as chemical potential. This shows the increasing order of the electrophilic character in the element, based on the extent of their electrophilic value^54^. The electrophilic characters of the AEET compound are represented in Table 3, which matches with their LUMO electrophilic energy values. Furthermore, the ability of molecules to donate electrons is related to nucleophilicity character. Therefore, the large nucleophilicity power refers to strong nucleophilic features^54,55^. Nucleophilicity results, which are opposite to electrophilicity and refer to the nucleophilic power as in Table 3, can also refer to the LUMO values. As for the liquid phase, that indicates the results are the same object represented in HOMO and LUMO values, which means any increase from compound power to accept electrons decreases the ability to lose electrons in the same compound. Corrosion studies include the (ΔN) parameter, which refers to the electrons transferred fraction and can be mentioned in Eq. 14.

14 $$\:\varDelta\:N=\frac{[\phi\:-\:{\chi\:\:}_{inh}]}{\left[2\right({\eta\:}_{Fe}\:+\:{\eta\:\:}_{inh}]\:\:\:}$$

So that, the parameters (φ) correspond to the function of work index, (χ inh) is the inhibitor electronegativity, (η inh) is the inhibitor hardness, and (η Fe) is the hardness of the Fe element metal. It’s important to note that the 4.82 eV refer to the values of work function for the simulated Fe (110) metal surface, while the hardness of iron can be reported as zero.

The (ΔN) parameter indicates the molecule’s ability to transfer electrons to a metal surface. So, it is possible to transfer the electron from the metal surface in the present inhibitors molecules if ΔN > 0, and conversely, the transfer can take place from the metal to the inhibitor if ΔN < 0^57^.

**Table 3:** Quantum chemical parameters of the AEET compound in liquid and gas phases.

Parameter	Phase	
	Gas	Liquid
E-_HOMO_	− 4.821	− 5.073
E-_LUMO_	− 1.568	− 1.847
∆E	3.253	3.223
η	1.626	1.611
Χ	3.194	3.458
µ _(Debye)_	5.765	9.345
Ω	3.545	5.800
Ε	0.282	0.172
σ, (eV-1)	0.615	0.620
ΔN	0.510	0.422

#### Molecular dynamic simulations

**Fig. 9:**
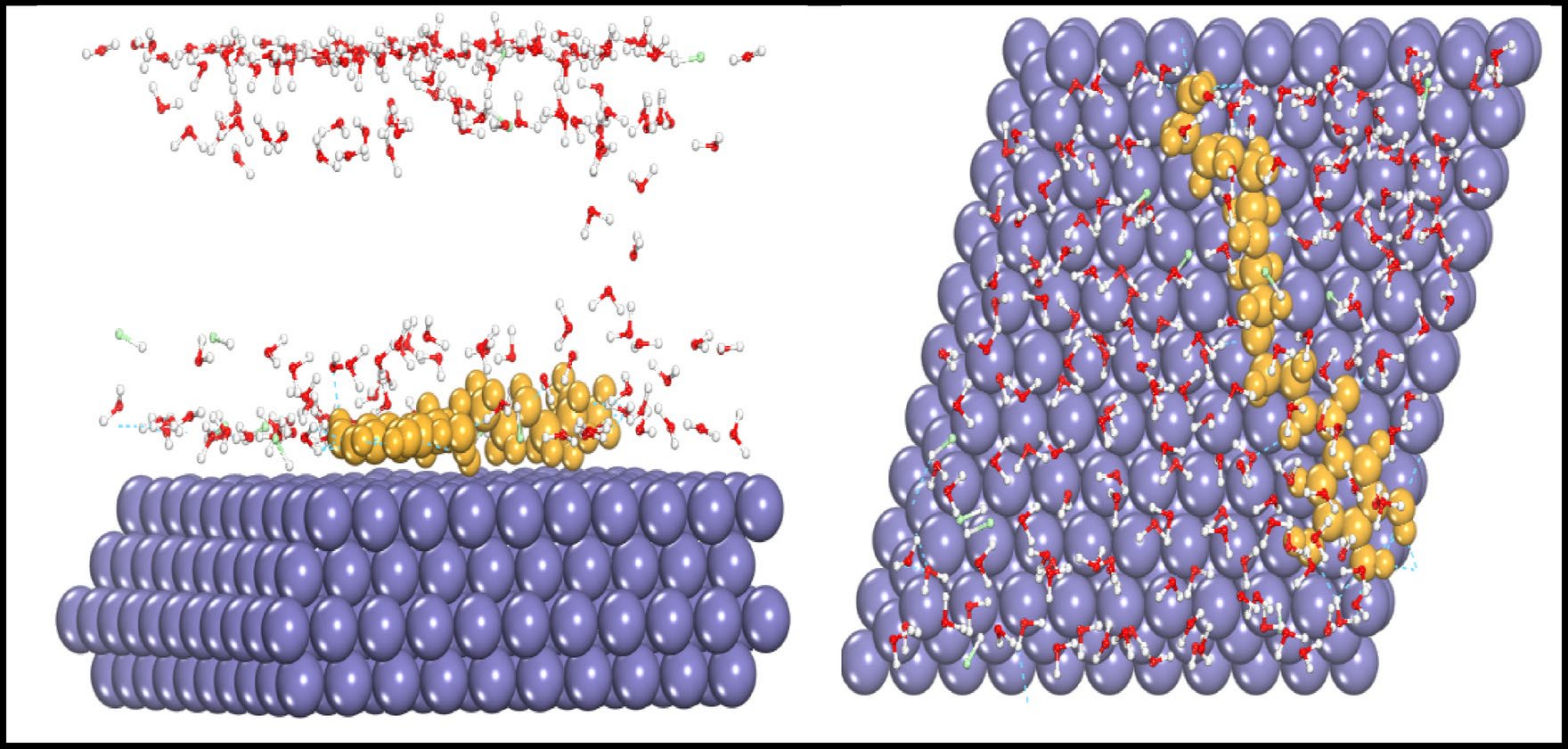
Both of side (left) and top (right) dimensioned views of AEET (yellow color) located barrel on iron atoms (110) surface in the box of water and HCl molecules.

Quantum chemical calculations describe the physical and chemical properties of an inhibitor molecule. Although the information collected is useful in understanding inhibitor reactions with water, there are limits to studying inhibitor reactions with a simulated metal surface (MS). To address these constraints, researchers have lately attempted to employ molecular dynamic simulations to investigate the behavior of inhibitor molecules on a simulated metal surface in order to detect physical adsorption with a solvent^57,58^. Figure 9 depicts the adsorption arrangements of the AEET compound on the Fe (110) surface (side and top views). It should concentrate on the primary function groups that have a strong interaction force with inhibitor molecules, which include active function groups such heteroatom chains, imine groups, the power of aromatic ring resonances, and the diverse dimensional positions of molecules (Table 4).

**Table 4:** The observed energies calculated by (MC) simulation for (AEET) on Fe (110) surface.

Compound	Adsorption energy (kJ mol^− 1^)	Rigid adsorption energy (kJ mol^− 1^)	Deformation energy (kJ mol^− 1^)	(dE_ads_/d_Ni_) (kJ mol^− 1^)	(dE_ads_/d_Ni_) (kJmol^− 1^ ).WTR
Gas phase					
AEET	− 195.16	− 198.06	2.89	− 195.16	–
Liquid phase (180part.H2O)					
AEET	− 2340	− 2453.99	113.99	–	− 11.25

The observed results indicate that the prepared inhibitor molecules were adsorbed on the iron surface and lying in a parallel arrangement after data analysis. The parallel arrangement will unquestionably provide evidence of the powerful interactions that exist between function groups that are contained inside the same molecules and that cover the greatest portion of the surface of the simulated iron atom. As an additional feature, Fig. 9 provides a description of the dynamic geometries of the simulated inhibitor molecules that were adsorbed on the surface of Fe (110) in a variety of dimensions. According to the findings that have been reported, the adsorption of the inhibitor molecules is extremely near to that of the neutral molecules when the orientated molecules are slightly parallel to one another. In addition to neutral molecules, it appears that the liquid-phase molecule possesses a high potential for preventing corrosion and may offer robust protection for the surface of the iron^10,26–59^.

Another method for identifying molecular interactions and binding energies, once the adsorption system reaches equilibrium, is important for understanding the interactivity of the molecules^60^. The interaction and binding energies of the molecule in the gas and liquid phases are presented in Table 5 below. An inhibitor molecule exhibiting high binding energy indicates both a strong interaction with the Fe(110) surface and enhanced adsorption of the inhibitor molecules onto the iron surface^59,60^. The results in Table 4 indicate that the AEET molecule exhibits significant binding energies in its adsorption performance, suggesting that the inhibitor molecule forms multiple bonds with the iron surface^60^. The results align with DFT and experimental studies discussed in this paper and others, indicating that electron-donating functional groups may enhance the corrosion inhibition process. The findings indicate that the AEET inhibitor exhibits a high adsorption capacity and functions effectively as an inhibitor^61^. AEET molecules exhibit greater binding and interaction energies in their liquid phase compared to their gas phase form. This is due to the increased reactivity of these compounds in liquid form, as explained by the DFT tool. The primary reason for these differences is the inhibitor molecule’s capacity to donate electrons. During the inhibition process, notable interactions between gas and liquid molecules and iron atoms are expected^62^.

**Table 5:** Binding and interaction energies of the AEET compound adsorbed on simulated iron atoms (110) surface.

AEET	Binding energy	Interaction energy
	Gas phase	
	− 241.06	− 201.33
	Solution phase	
	− 805.91	− 865.82

#### RDF’s analysis

The radial distribution function (RDF) is a concept that illustrates how the density of matter in the surrounding area changes as one moves further away from a specific location. It gives us information about the frequency with which particular distances were recorded. When performing an MD simulation, the RDF is calculated by counting the number of atom pairs that are located between the gaps that have been set. The findings can be applied to the average atomic density as a function of distance, which is an objective that is even achievable.

**Fig. 10:**
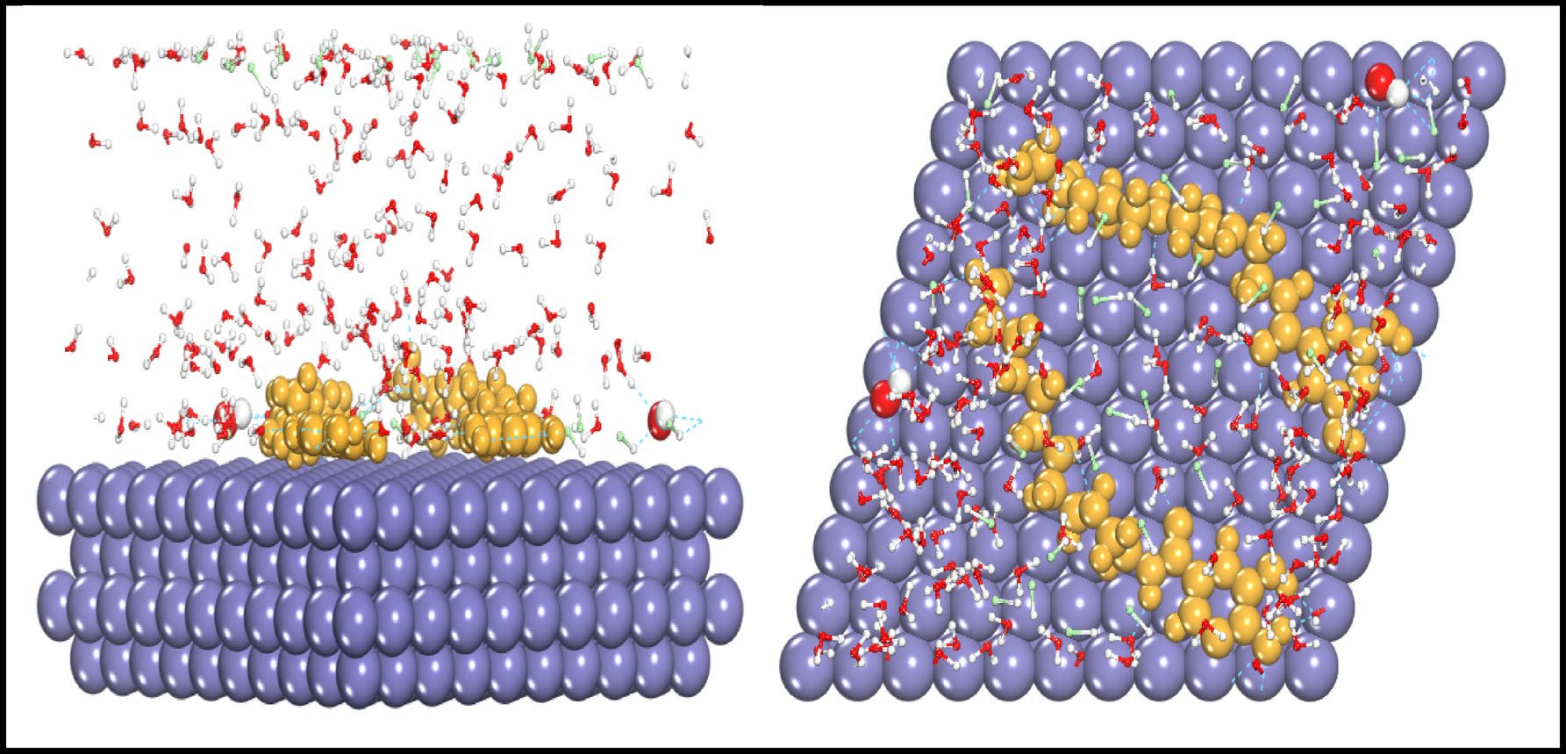
Both of side (left) and top (right) dimensioned views of two AEET (yellow color) located barrel on iron atoms (110) surface in the box of water and HCl molecules.

**Fig. 11:**
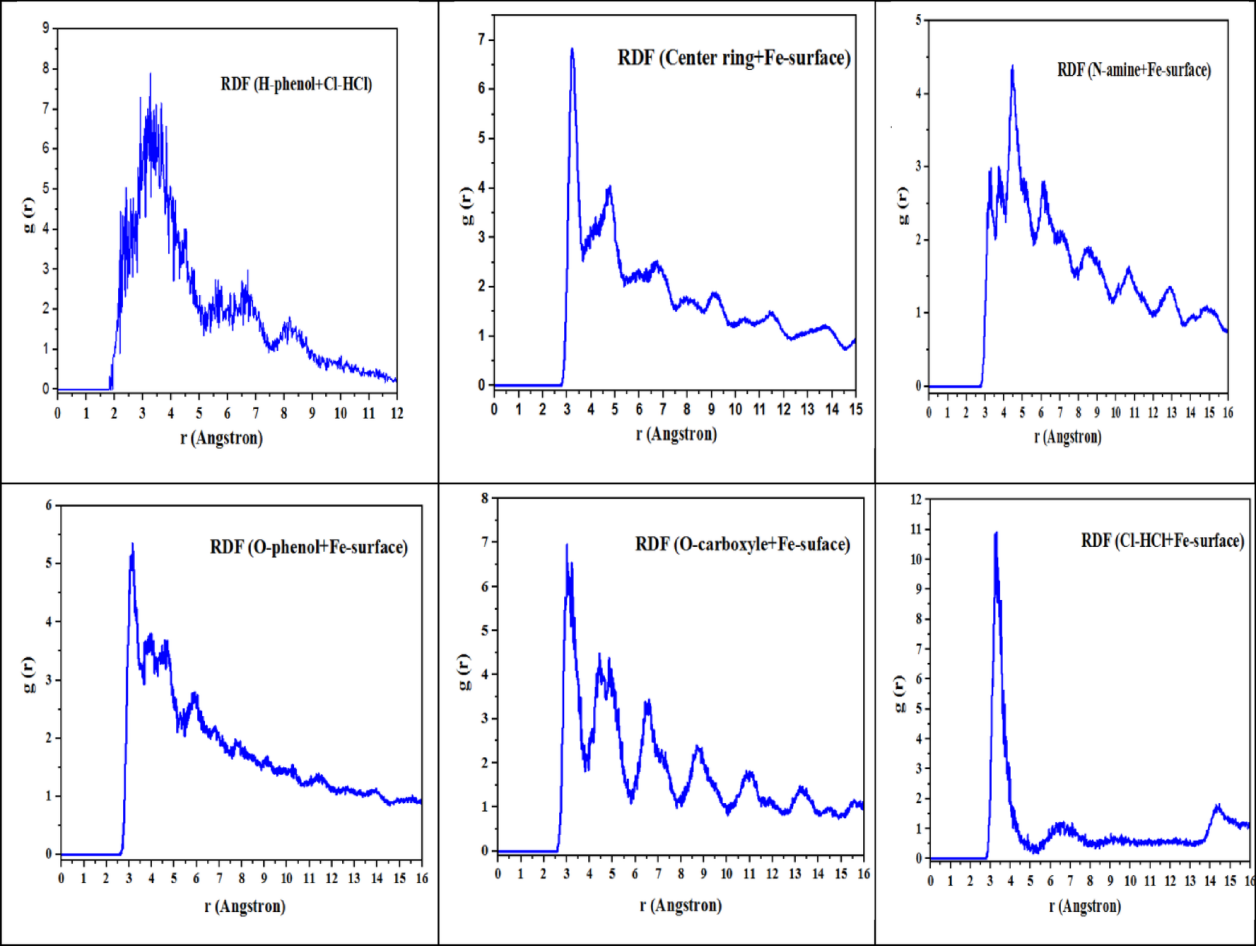
RDF analysis for AEET function groups interact with Fe-surface and environmental surround the molecules.

The additional outcome was the calculation of the Radial Distribution Function (RDF), which aids in identifying the position in space between atoms and molecules, as well as the associated contact and binding energies. Molecules positioned at distances ranging from 1 Å to 3.5 Å are likely to get close to iron atoms and establish covalent bonds with adjacent atoms, whereas those separated by distances exceeding 3.5 Å can only engage in physical interactions^63^. The RDF findings indicated that the bonding lengths between the ring atoms, Fe-surface, and O-carboxyl, phenol-Fe-surface were below 3.5 Å. In contrast, the bonding lengths between N-amine-Fe-surface exhibited a broader peak, approaching 3.5 Å. This suggests that the interactions between iron atoms and oxygen, amines, and carbon ring atoms were effectively observed during analysis^62–64^. This paper presents calculations of radial distribution functions (RDF) to construct two simulation scenarios involving two cells. The first cell contains a (AEET) molecule in a box with H2O particles and corrosive ions (Cl- and H3O+) directly interacting with the iron atoms (Fe) surface to evaluate the interaction energy and RDF, as illustrated in Fig. 9. The second scenario involves two (AEET) molecules within the same box to simulate the interaction between these inhibitor molecules and their masking effect on the iron surface, as well as their influence on the distribution of corrosive particles, also depicted in Fig. 10. To optimize simulation processes, adjust the NVT and COMPASS force field to align with the calculated Van der Waals and electrostatic interactions using the Ewald summation technique and atom-based selection. A simulation duration of 1000 ps was conducted to obtain results regarding the (AEET) inhibitor within the simulated system^62^. At the conclusion of the simulations, the (AEET) molecules exited the solvent and aligned parallel to the iron surface, indicating a strong interaction with iron atoms and covering a significant portion of the iron surface. RDF results indicated that functional groups, including O, NH₂, and C atoms, within the ring are positioned approximately 3.5 Å apart. The formation of covalent bonds with Fe atoms is probable when compared to the RDF depicting the Cl-Fe surface, which exhibits a weak bond attributed to OH⁻ groups that hinder ion access to the iron surface during our simulated cell, as illustrated in Fig. 11.

#### Antimicrobial activity of AEET against SRB

**Fig. 12:**
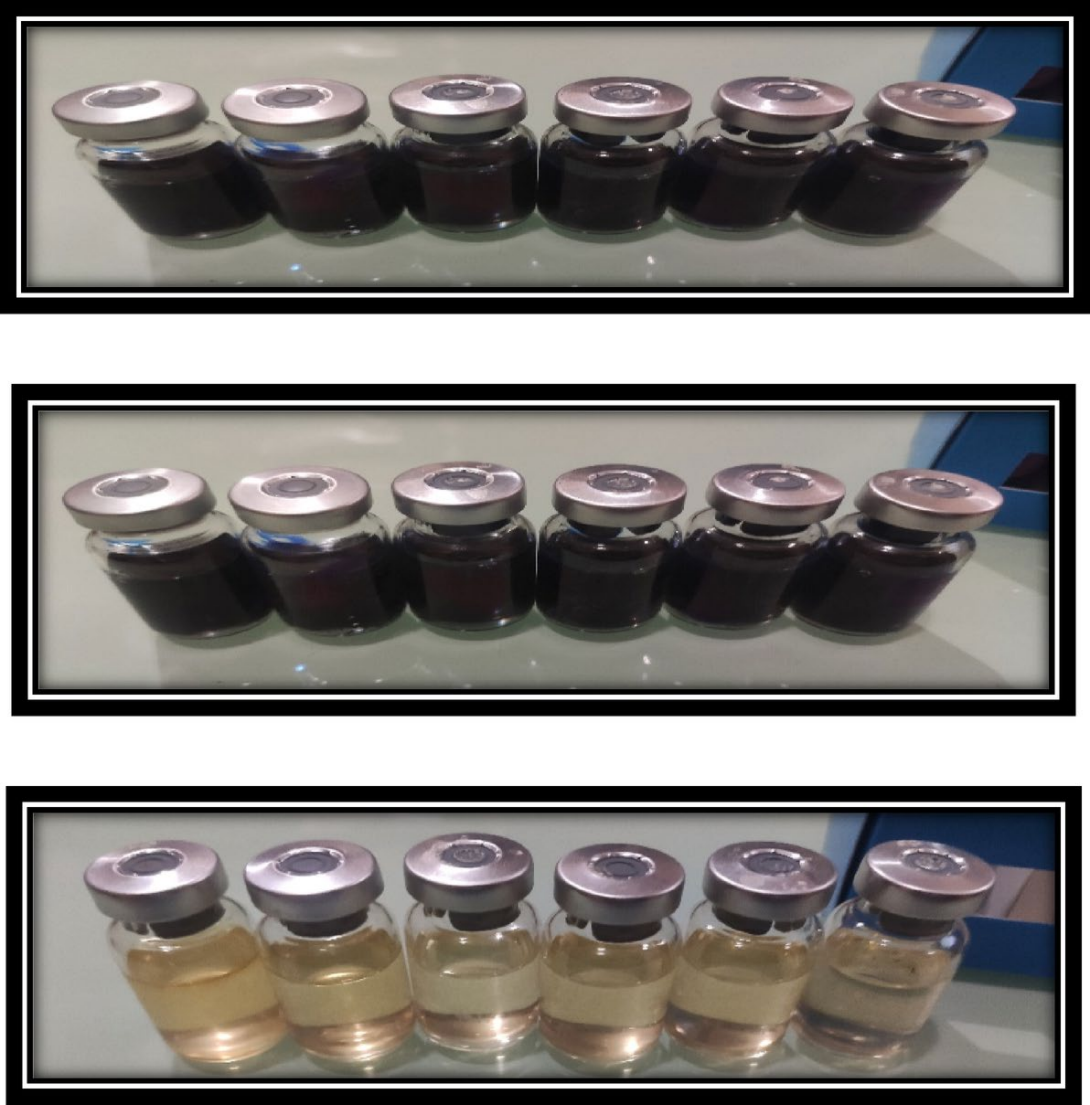
SRB growth measurement at various concentrations of infected water samples.

The number of microbial cells related to SRB that is present in the water sample after undergoing the interaction with the AEET molecule is depicted in Fig. 12. These images represent the sealed glass vials maintained under low oxygen conditions. Concentration serials of the inhibitor at 1 × 10^− 4^ M and 2 × 10^− 4^ M were added into the vials. Figure 9 presents images of the vial sets injected with a blank for comparison. The number of black vials (infected) was significantly lower than the blank vials, which proves the biocidal efficiency^65,66^. Black vials were evidently scanty as compared to the white vials, suggesting the efficiency of the black vials against this strain of bacteria^67,68^.

Table 6 elucidates the data derived from the antimicrobial activity test of the tested biocides against SRB growth. Notably, the counts returned were 102 cells/ml and 0 cells/ml in the 1 × 10^− 4^ M and 2 × 10^− 4^ M of AEET, respectively, proof that AEET offered the best inhibitor activity in preventing bacterial growth, achieving up to a 100% reduction (Table 6). Biocidal efficacy of AEET against SRB at 40 °C.

**Table 6:** Media kits of SRB growth injected with infected water samples with different concentrations of AEET.

	Dose (M)	SRB count (cell/ml)	Reduction in SRB count (cell/ml)	Efficiency %
Blank	–	10^6^	–	–
AEET	1 × 10^− 4^	10^2^	10^4^	66.7
	2 × 10^− 4^	10^0^	10^6^	100

#### Docking analysis

**Fig. 13:**
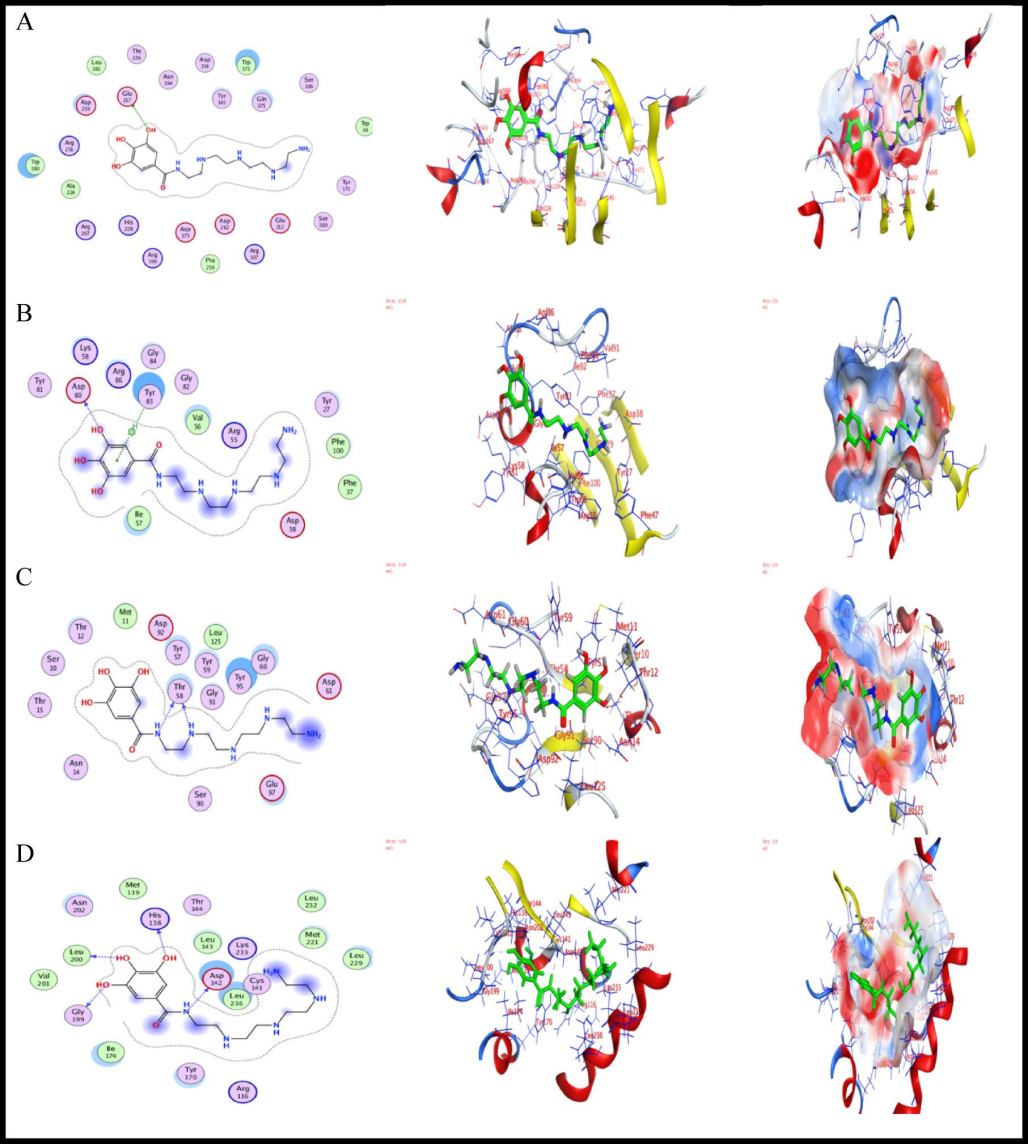
The crystal structures of protein targets describe the AEET at large pocket (site-1) in the active pocket of (**A**) 4ZZT, (**B**) 5HWC, (**C**) 5LJI and (**D**) 3T88.

**Fig. 14:**
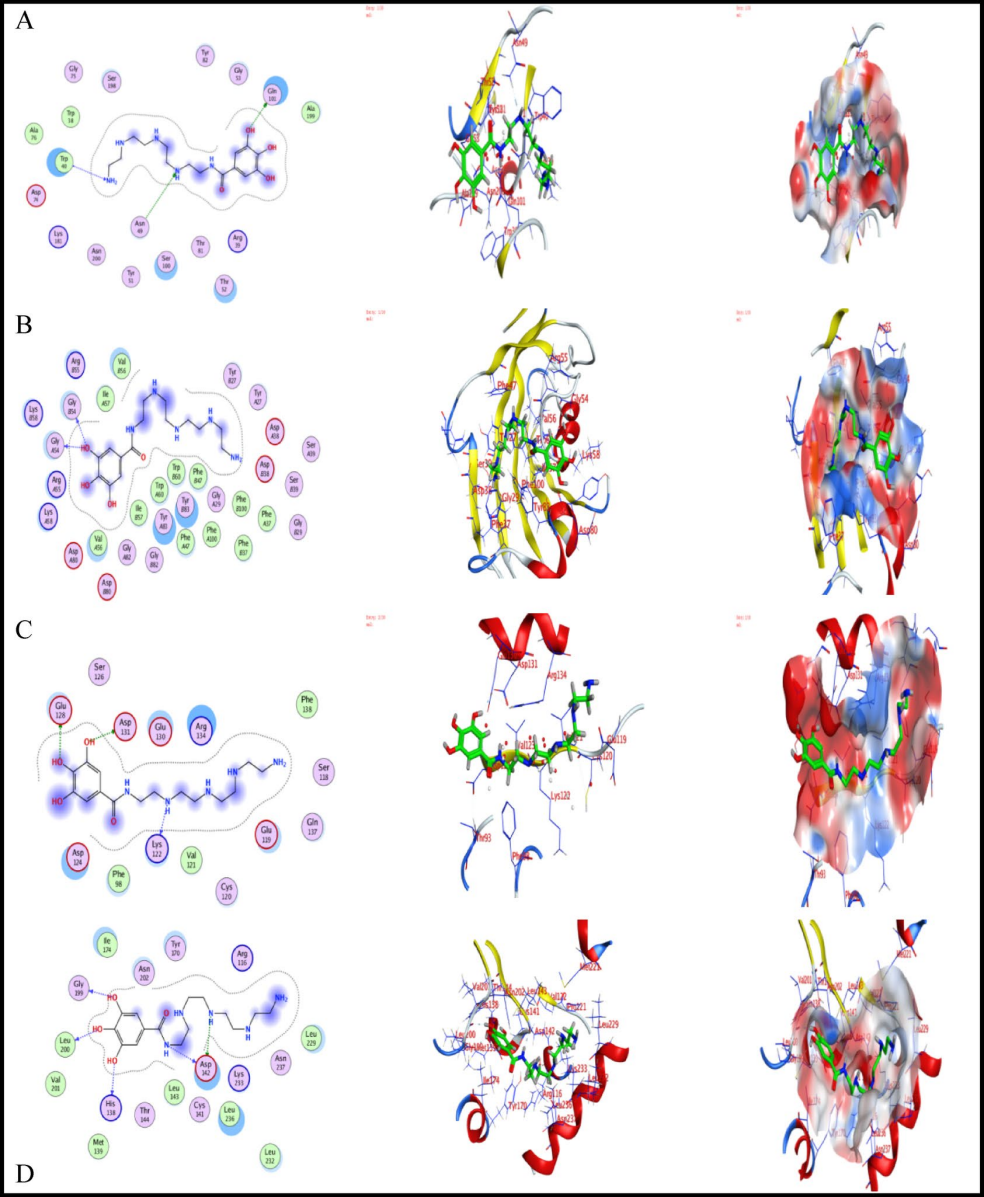
The crystal structures of protein targets describe the AEET at large pocket (site-2) same site of original legend in the active pocket of (**A**) 4ZZT, (**B**) 5HWC, (**C**) 5LJI and(D) 3T88 .

Docking studies were conducted to elucidate the biological activity outcomes of AEET-tested compounds through the interaction between ligand and protein receptors. This paper presents a computational docking analysis of active pocket sites in various crystal protein structures sourced from the Protein Data Bank. The analysis aims to align with the experimental results obtained to address the SRB issue. The bacterial strain structures were selected based on their prevalence in industrial applications: A. fumigatus (ID 5HWC)^69^, G. candidum (ID 4ZZt)^70^, S. pneumoniae (ID 5LJI)^71^, and E. coli (ID 3t88)^72^. These were imported into MOE in 3D structure and energy minimized to a gradient of 0.01. The compound AEET was imported in MDB format for simulation with the selected bacteria. Issues with protein structure were corrected, and atoms were protonated in their 3D geometric structures after the removal of water and solvent particles. The site finder option was then utilized to identify the most suitable amino acids for reaction at various active sites, as detailed in Table 7. Figures 12 and 13 present the docking computations and crystal structures simulated using the Triangle matcher and GBVI/WSA dG refinement methods^73^. Following the docking process, 10 poses were selected from a total of 200 based on the most favorable score and RMSD, and subsequently adjusted for the export of interaction energy^74^. Interaction energies with the specified amino acids of the residue pockets are presented in Tables 8 and 9. The study presented an alternative approach to understanding electrostatic interactions with protein active sites^75–78^. The AEET molecules exhibited acceptable RMSD values near the original legend site-1 across most receptors; however, crystal structures 5LJI and 3T88 displayed identical binding positions to the original with the AEET inhibitor. The 5LJI residue demonstrated binding interactions with the AEET compound, achieving a score of − 6.36 and an RMSD of 1.38, as illustrated in Fig. 13. The hydrogen bonds are associated with GLU-128 (3.02 Å), indicating a significant binding interaction. The binding interactions of 3T88 with the AEET compound yield a score of − 6.99 and an RMSD of 1.7,as illustrated in Fig. 13. Additionally, hydrogen bonds are formed with GLY-199 at a distance of 2.76 Å, indicating a significant binding interaction^72^. Our study demonstrates that the AEET inhibitor exhibits biological activity against the selected bacteria, achieving promising binding scores Fig. 14. through the formation of extensive hydrogen bonds with energies of up to − 7 kcal/mol. AEET demonstrates superior binding scores with receptors 4ZZT, 5LJI, 3T88, and 5HWC (− 8.48, − 7.71, − 7.12, and − 5.10, respectively) compared to site 2, the original ligand (− 6.9). This computational analysis aligns with the previously mentioned experimental results, indicating that AEET molecules exhibit notable biological activity against SRB, which contributes to corrosion in mild steel.

**Table 7:** Binding sites residues extracted from active pocket receptor of 4ZZT, 5HWC, 5LJI, 3T88 during prepared for docking.

Receptors	Site	Residues
4ZZT	1	1:(THR7 LEU34 ASN37 TRP38 ARG39 TRP40 TYR51 THR52 GLY53 ASP74 GLY75 ALA76 ASP77 THR81 TYR82 SER100 GLN101 LYS102 ASN103 VAL104 GLY105 SER106 ARG107 TYR145 TYR171 ASP173 SER174 GLN175 ASP179 LYS181 PRO194 ALA195 SER196 SER198 ALA199 ASN200 SER201 GLY202 LEU203 GLU212 ASP214 GLU217 ALA224 THR226 HIS228 THR246 TYR247 ARG251 TYR252 PRO258 ASP259 ASP262 ASN264 ARG267 LYS338 PHE341 GLY342 ASP343 THR344 SER369 TRP371 ASP372 ASP373 TYR374 TYR375 TRP380 LEU381 TYR386 PRO387 ARG399 GLU413)
	2	1:(ASN49 TRP38 ARG39 TRP40 ASN49 TYR51 THR52 GLY53 GLN101 LYS181 ALA199 ASN200)
5HWC	1	1:(TYR27 PHE37 ASP80 ARG43 PHE47 ARG55 VAL56 ILE57 TRP60 TYR83 PHE88 VAL91 ILE92 PHE100)
	2	1:(LYS8 HIS26 TYR27 THR28 VAL41 G LY54 ALA73 GLU101 VAL102 GLU103)
5LJI	1	1:(ASN14 TYR57 THR58 TYR59 GLY60 SER90 GLY91 ASP92 TYR95 GLU97 PHE98 CYS99 LEU125)
	2	1:(THR93 PHE98 GLU119 CYS120 VAL121 LYS122 VAL123 ASP124 GLU128 ASP131 ARG134)
3T88	1	1:(VAL44 ARG45 ALA47 PHE48 SER84 GLY85 GLY86 ASP142 GLN88 LYS89 ARG91 TYR97 LEU106 VAL108 LEU109 GLN112 TYR129 ILE131 GLY132 GLY199 GLN154 THR155 GLY156 VAL159 SER161 PHE162 ASP163 GLY164 GLY165 TRP184 GLN189)
	2	1:(ARG116 PRO121 HIS138 MET139 MET140 CYS141 ASP142 LEU143 THR144 TYR170 ARG173 ILE174 GLY133 LEU200 VAL201 ASN202 MET221 LEU229 LEU232 LYS233 LEU236 ASN237 CYS240)

**Table 8:** The finding extracted from Docking process of AEET with receptors in site-1 the large pocket of residues.

Ligand	Receptor	S score (kcal/mol)	RMSD (Å)	Bonds type among atoms of AEET and residues of active sites of receptors					
				AEET atoms	Atom receptor	receptor residues	Interaction bond type	Distance (Å)	E_int_ (kcal/mol)
AEET	4ZZT	− 8.486	1.504	O-13	OE-2	GLU-217	H-donor	2.68	− 3.1
	5HWC	− 6.210	1.716	O-13	O	ASP-80	H-donor	2.97	− 3.0
				6-ring	CA	TYR-83	Pi-H	3.72	− 0.6
	5LJI	− 7.711	1.388	N-16	O	THR-58	H-donor	2.98	− 3.6
				N-24	O	THR-58	H-donor	2.96	− 0.9
	3T88	− 7.121	1.776	O-9	O	HIS-138	H-donor	3.13	− 0.8
				O-11	O	LEU-200	H-donor	2.85	− 1.8
				O-13	O	GLY-199	H-donor	2.74	− 1.4
				N-16	O	ASP-142	H-donor	2.94	− 2.9

**Table 9:** The finding extracted from Docking process of AEET with receptors in site-2(original ligand site).

Ligand	Receptor	S score (kcal/mol)	RMSD (Å)	Bonds type among atoms of AEET and residues of active sites of Receptors					
				AEET atoms	Atom receptor	receptor residues	Interaction bond Type	Distance (Å)	Eint (kcal/mol)
AEET	4ZZT	− 6.554	2.054	O-13	OE-1	GLN-101	H-donor	3.10	− 1.0
				N-24	ND-2	ASN-49	H-acceptor	3.31	− 2.5
				N-48	N	TRP-40	H-acceptor	3.25	− 3.2
	5HWC	− 6.210	1.836	O-13	O	GLY-54	H-donor	2.94	− 2.3
				O-13	O	GLY-54	H-donor	2.94	− 2.3
	5LJI	− 6.364	1.391	O-11	OE-2	GLU-128	H-donor	3.02	− 1.4
				O-13	OD-2	ASP-131	H-donor	3.32	− 1.3
				O-24	O	LYS-122	H-donor	3.01	− 0.9
	3T88	− 6.997	1.768	O-9	O	HIS-138	H-donor	3.10	− 1.0
				O-11	O	LEU-200	H-donor	2.84	− 1.9
				O-13	O	GLY-199	H-donor	2.76	− 1.6
				N-16	O	ASP-142	H-donor	3.06	− 2.5
				N-32	OD-1	ASP-142	H-donor	2.85	− 1.0

## Conclusion

This study offers experimental and computational analyses to demonstrate the efficiency of the eco-friendly Schiff base compound AEET as a multifunctional corrosion inhibitor for mild steel in 1 M HCl. Electrochemical data demonstrates a concentration-dependent improvement in inhibition efficiency, with IE% increasing from 48.08% at 25 ppm to 90.05% at 5 × 10^− 4^. A big rise in polarization resistance (Rp: 176.02 to 2593.93 Ω.cm) and a drop in corrosion rate (CR: 6.2 to 0.62 mpy) show that this is true. The observed changes validate the formation of a protective AEET layer on the metal surface, as evidenced by SEM images. Quantum chemical calculations using DFT demonstrate AEET’s electronic suitability for adsorption, characterized by a narrow HOMO-LUMO energy gap (ΔE ≈ 3.2 eV) and a high adsorption energy (-805.91 kJ/mol in solution phase), which suggest strong electron donation and stable binding to the metal surface. Molecular docking demonstrates AEET’s capacity to interfere with microbial corrosion mechanisms, as indicated by significant binding affinities (S scores: -6.2 to -8.5 kcal/mol) with corrosion-related receptors (e.g., SRB enzymes) through hydrogen bonds (2.68–3.72 Å), which reduce microbial activity. Computational simulations (MD, MC) support experimental findings, demonstrating the multifunction inhibition mechanism through the formation of a dense protective barrier that obstructs aggressive ions such as Cl⁻ and H⁺ and the selective disruption of microbial enzymes to mitigate microbiologically influenced corrosion (MIC). The combination of experimental measurements, such as SEM and electrochemical parameters, with theoretical models including DFT, docking, and simulations, highlights the reliability of AEET as a cost-effective and environmentally friendly inhibitor. This integrated strategy connects molecular-level interactions, such as adsorption energy and bond distances, to macroscopic performance, establishing AEET as a useful industrial solution for addressing both chemical and microbial corrosion.

## Data Availability

All data generated or analyzed during this study are included in this article.

## References

[1] Panossian, Z., Almeida, N. L. D., Sousa, R. M. F. D., Pimenta, G. D. S. & Marques, L. B. S. Corrosion of carbon steel pipes and tanks by concentrated sulfuric acid: a review. *Corros. Sci.* **58**, 1–11 (2012).

[2] Finšgar, M. & Jackson, J. *Application of Corrosion Inhibitors for Steels in Acidic Media for the Oil and Gas Industry: A review*, 17–14 (Science Direct, 2014).

[3] Osman, M. M. & Shalaby, M. N. Some ethoxylated fatty acids as corrosion inhibitors for low carbon steel in formation water. *Mater. Chem. Phys.* **77**, 261–269 (2003).

[4] Ghareba, S. & Omanovic, S. Interaction of 12-aminododecanoic acid with a carbon steel surface: towards the development of ‘green’ corrosion inhibitors. *Corros. Sci.* **52**, 2104–2113 (2010).

[5] Wilhelm, S. M. Galvanic corrosion in oil and gas production: part 1—laboratory studies. *Corrosion* **48**, 691–703 (1992).

[6] Martínez, D. et al. Amine type inhibitor effect on corrosion–erosion wear in oil gas pipes. *Wear* **267**, 255–258 (2009).

[7] Abd El-Lateef, H. M., Abu-Dief, A. M. & Mohamed, M. A. A. Corrosion inhibition of carbon steel pipelines by some novel schiff base compounds during acidizing treatment of oil wells studied by electrochemical and quantum chemical methods. *J. Mol. Struct.* **1130**, 522–542 (2017).

[8] Zhai, W., Bai, L., Zhou, R., Fan, X., Kang, G., Liu, Y., & Zhou, K. Recent progress on wear‐resistant materials:designs, properties, and applications. *Adv. Sci.* **8**(11), 2003739 (2021). 10.1002/advs.202003739 PMC8188226 34105292

[9] Ituen, E. B., Solomon, M. M., Umoren, S. A. & Akaranta, O. Corrosion inhibition by amitriptyline and amitriptyline based formulations for steels in simulated pickling and acidizing media. *J. Pet. Sci. Eng.* **174**, 984–996 (2019).

[10] Abbas, M. A. et al. Multifunctional aspects of the synthesized pyrazoline derivatives for AP1 5L X60 steel protection against MIC and acidization: electrochemical, in Silico, and SRB insights. *ACS Omega*. **6**, 8894–8907 (2021). 33842760 10.1021/acsomega.0c06050 PMC8028000

[11] Eid, A. M., Shaaban, S. & Shalabi, K. Tetrazole-based organo- selenium bi-functionalized corrosion inhibitors during oil well acidizing: experimental, computational studies, and SRB bioassay. *J. Mol. Liq*. **298**, 111980 (2020).

[12] Ansari, K. R., Chauhan, D. S., Singh, A., Saji, V. S. & Quraishi, M. A. Corrosion inhibitors for acidizing process in oil and gas sectors. In *Corrosion Inhibitors in the Oil and Gas Industry*, 151–176. ( Wiley, 2020).

[13] Zhu, Y., Free, M. L. & Yi, G. Electrochemical measurement, modeling, and prediction of corrosion inhibition efficiency of ternary mixtures of homologous surfactants in salt solution. *Corros. Sci.* **98**, 417–429 (2015).

[14] Chigondo, M. & Chigondo, F. Recent natural corrosion inhibitors for mild steel: an overview. *J. Chem.* **2016**(1), 6208937 (2016).

[15] Oguzie, E. E. et al. Natural products for materials protection: mechanism of corrosion Inhibition of mild steel by acid extracts of Piper guineense. *Phys. Chem. C*. **116**, 13603–13615 (2012).

[16] Abdallah, M., Al-Tass, H. M., Jahdaly, A. L. & Fouda, B. A. Inhibition properties and adsorption behavior of 5-Arylazothiazole derivatives on 1018 carbon steel in 0.5 M H_2_SO_4_ solution. *J. Mol. Liq*. **216**, 590–597 (2016).

[17] Shaban, M. M. et al. Novel trimeric cationic pyrdinium surfactants as bi-functional corrosion inhibitors and antiscalants for API 5L X70 carbon steel against oilfield formation water. *J. Mol. Liq*. **305**, 112817 (2020).

[18] Wang, J. et al. The performance and mechanism of bifunctional biocide sodium pyrithione against sulfate reducing bacteria in X80 carbon steel corrosion. *Corros. Sci.* **150**, 296–308 (2019).

[19] Kavitha, K., Benitasherine, H. & Rajendran, S. Cardiospermum Halicacabum leaves extract as a green corrosion inhibitor for mild steel in simulated oil well water medium. *Int. J. Corros. Scale Inhib.* **10**(4), 1646–1660 (2021).

[20] Fouda, A. S., Eldesoky, A. M., Diab, M. A. & Nabih, A. Inhibitive, adsorption studies on carbon steel corrosion in acidic solutions by new synthesized benzene sulfonamide derivatives. *Int. J. Electrochem. Sci.* **11**, 9998–10019 (2016).

[21] Sapkota, K. et al. Antioxidant and antimelanogenic properties of chestnut flower extract. *Biosci. Biotechnol. Biochem.* **74**, 1527–1533 (2010). 20699587 10.1271/bbb.100058

[22] Santamaria, S. et al. N-O-isopropyl sulfonamido-based hydroxamates: kinetic characterisation of a series of MMP-12/MMP-13 dual target inhibitors. *Biochem. Pharmacol.* **84**, 813–820 (2012). 22771631 10.1016/j.bcp.2012.06.026

[23] Hsiang, C. Y. et al. Toona sinensis and its major bioactive compound gallic acid inhibit LPS–induced inflammation in nuclear factor–kappaB Transgenic mice as evaluated by in vivo bioluminescence imaging. *Food Chem.* **136**, 426–434 (2013). 23122080 10.1016/j.foodchem.2012.08.009

[24] Ovung, A. & Bhattacharyya, J. Sulfonamide drugs: structure, antibacterial property, toxicity, and biophysical interactions. *Biophys. Rev.* **13**, 259–272 (2021). 33936318 10.1007/s12551-021-00795-9 PMC8046889

[25] Yi, F., Wu, K., Yu, G. & Su, C. Preparation of Pickering emulsion based on soy protein isolate-gallic acid with outstanding antioxidation and antimicrobial. *Colloid Sand Surf. B: Biointerfaces*. **206**, 111954 (2021). 10.1016/j.colsurfb.2021.111954 34229175

[26] Badhani, B., Sharma, N. & Kakkar, R. Gallic acid: a versatile antioxidant with promising therapeutic and industrial applications. *RSC Adv.* **5**, 27540–27557 (2015).

[27] El Basiony, N. M., Elgendya, A., Nadyb, H. & Migaheda, M. A. Zaki adsorption characteristics and inhibition effect of two schiff base compounds on corrosion of mild steel in 0.5 M HCl solution: experimental, DFT studies, and Monte Carlo simulation. *RSC Adv.* **9**, 10473–10485 (2019). 35515280 10.1039/c9ra00397e PMC9062527

[28] Bedair, M. A. et al. Benzidine-based schiff base compounds for employing as corrosion inhibitors for carbon steel in 1.0 M HCl aqueous media by chemical, electrochemical and computational methods. *Biointerfaces*10.1016/j.molliq.2020.114015 (2021).

[29] Salhi, A. et al. A correlated theoretical and electrochemical investigation of the corrosion Inhibition performance of phenolic schiff bases on mild steel in HCl solution (Part B). *Inorganic Chem. Commun.* **152**, 110684 (2023).

[30] Metwally, M. A., Bondock, S., El-Desouky, E. S. I. & Abdou, M. M. A facile synthesis and tautomeric structure of novel 4-arylhydrazono-3-(2-hydroxyphenyl)-2-pyrazolin-5-ones and their application as disperse dyes. *Color. Technol.* **129**, 418–424 (2013).

[31] El Defrawy, A. M., Abdallah, M. & Al-Fahemi, J. H. Electro- chemical and theoretical investigation for some pyrazolone derivatives as inhibitors for the corrosion of C-steel in 0.5 M hydrochloric acid. *J. Mol. Liq*. **288**, 110994 (2019).

[32] Geng, W., Yuan, Q., Jiang, X., Tu, J., Duan, L., Gu, J., & Zhang, Q. Humidity sensing mechanism of mesoporous MgO/KCl–SiO2 composites analyzed by complex impedance spectra and bode diagrams. *Sens. Actuators B: Chem.* **174**, 513–520 (2012).

[33] Wu, P. P. et al. Effect of duty cycle on preparation and corrosion behavior of electrodeposited calcium phosphate coatings on AZ91. *Appl. Surf. Sci.* **426**, 418–426 (2017).

[34] Abd-Elaal, A. A., Elbasiony, N. M., Shaban, S. M. & Zaki, E. G. Studying the corrosion inhibition of some prepared nonionic surfactants based on 3-(4-hydroxyphenyl) propanoic acid and estimating the influence of silver nanoparticles on the surface parameters. *J. Mol. Liq*. **249**, 304–317 (2018).

[35] Migahed, M. A., Zaki, E. G. & Shaban, M. M. Corrosion control in the tubing steel of oil wells during matrix acidizing operations. *RSC Adv.* **6**, 71384 (2016).

[36] Abbas, M. A. & Bedair, M. A. Adsorption and computational studies for evaluating the behavior of silicon based compounds as novel corrosion inhibitors of carbon steel surfaces in acidic media. *Z. Physiol. Chem.* **233**, 225–254 (2019).

[37] Migahed, M. A., El-rabiei, M. M., Nady, H., Gomaa, H. M. & Zaki, E. G. Corrosion inhibition behavior of synthesized imidazolium ionic liquids for carbon steel in deep oil wells formation water. *J. Bio- Tribo-Corrosion*. **3**, 22 (2017).

[38] Zhang, W. et al. Tetrahydroacridines as corrosion inhibitor for x80 steel corrosion in simulated acidic oilfield water. *J. Mol. Liq*. **293**, 111478 (2019).

[39] Migahed, M. A., Al-Sabagh, A. M., Khamis, E. A. & Zaki, E. G. Quantum chemical calculations, synthesis and corrosion Inhibition efficiency of ethoxylated-[2-(2-{2-[2-(2-benzenesulfonylamino-ethyl- amino)-ethylamino]-ethylamino}-ethylamino)-ethyl]-4-alkyl-benzene- sulfonamide on API X65 steel surface under H2S environment. *J. Mol. Liq*. **212**, 360 (2015).

[40] Hirschorn, B. et al. Constant-phase-element behavior caused by resistivity distributions in films: I. Theory. *J. Electrochem. Soc.* **157**, C452 (2010).

[41] Azghay, I. et al. Elucidating the corrosion Inhibition mechanisms: A computational and statistical exploration of the molecular structure-efficiency relationship for phenolic schiff bases in acidic medium on the mild steel surface. *J. Mol. Liq.* **393**, 123648 (2024).

[42] El Tamany, E. S. H., Elsaeed, S. M., Ashour, H., Zaki, E. G. & El Nagy, H. A. Novel acrylamide ionic liquids as anti-corrosion for X- 65 steel dissolution in acid medium: adsorption, hydrogen evolution and mechanism. *J. Mol. Struct.* **1168**, 106–114 (2018).

[43] Soliman, S. A., Metwally, M. S., Selim, S. R., Bedair, M. A. & Abbas, M. A. Corrosion inhibition and adsorption behavior of new schiff base surfactant on steel in acidic environment: experimental and theoretical studies. *J. Ind. Eng. Chem.* **20**, 4311–4320 (2014).

[44] Fouda, A. S., Zaki, E. G. & Khalifa, M. M. A. Some new nonionic surfactants based on propane tricarboxylic acid as corrosion inhibitors for low carbon steel in hydrochloric acid solutions. *J. Bio- Tribo-Corrosion*. **5**, 31 (2019).

[45] Ismail, A. S. & Abbas, M. A. The corrosion performance of Al-Si and Al-Cu casting alloys in H_2_SO_4_ and Na_2_CO_3_ media. *Silicon* **9**, 193–199 (2017).

[46] EL-Rabiei, M. M., Nady, H., Zaki, E. G. & Negem, M. Theoretical and experimental investigation of the synergistic influence of tricine and iodide ions on the corrosion control of carbon steel in sulfuric acid electrolyte. *J. Bio- Tribo-Corrosion*. **5**, 103 (2019).

[47] Zhai, W., Bai, L., Zhou, R., Fan, X., Kang, G., Liu, Y., & Zhou, K. Recent progress on wear‐resistant materials:designs, properties, and applications. *Adv. Sci.* **8**, 2003739 (2021). 10.1002/advs.202003739 PMC8188226 34105292

[48] Hegazy, M. A., Badawi, A. M., Abd, E., Rehim, S. S. & Kamel, W. M. Corrosion inhibition of carbon steel using novel N-(2-(2-mercaptoacetoxy)ethyl)-N, N-dimethyl dodecan-1-aminium bromide during acid pickling. *Corros. Sci.* **69**, 110–122 (2013).

[49] El Basiony, N. M., Elgendy, A., Nady, H., Migahed, M. A. & Zaki, E. G. Adsorption characteristics and inhibition effect of two schiff base compounds on corrosion of mild steel in 0.5 M HCl solution: experimental, DFT studies, and Monte Carlo simulation. *RSC Adv.* **9**, 10473–10485 (2019). 35515280 10.1039/c9ra00397e PMC9062527

[50] Hegazy, M. A., El-Tabei, A. S., Bedair, A. H. & Sadeq, M. A. An investigation of three novel nonionic surfactants as corrosion inhibitor for carbon steel in 0.5 M H_2_SO_4_. *Corros. Sci.* **54**, 219–230 (2012).

[51] Obot, I. B. & Obi-Egbedi, N. O. Adsorption properties and inhibition of mild steel corrosion in sulphuric acid solution by ketoconazole: experimental and theoretical investigation. *Corros. Sci.* **52**, 198–204 (2010).

[52] Musa, A. Y., Kadhum, A. A. H., Mohamad, A. B. & Takriff, M. S. Molecular dynamics and quantum chemical calculation studies on 4, 4-dimethyl-3-thiosemicarbazide as corrosion inhibitor in 2.5 M H_2_SO_4_. *Mater. Chem. Phys.* **129**, 660–665 (2011).

[53] Abyani, M. & Bahaari, M. R. A comparative reliability study of corroded pipelines based on Monte Carlo simulation and Latin hypercube sampling methods. *Int. J. Press. Vessels Pip.* **181**, 104079 (2020).

[54] Migahed, M. A., Al-Sabagh, A. M., Khamis, E. A. & Zaki, E. G. Quantum chemical calculations, synthesis and corrosion inhibition efficiency of ethoxylated-[2-(2-{2-[2-(2-benzenesulfonylamino-ethylamino)-ethylamino]-ethylamino}-ethylamino)-ethyl]-4-alkyl-benzenesulfonamide on API X65 steel surface under H2S environment. 10.1016/j.molliq.2015.09.032.

[55] Elaraby, A., El-Samad, S. A., Khamis, E. A. & Zaki, E. G. Theoretical and electrochemical evaluation of tetra-cationic surfactant as corrosion inhibitor for carbon steel in 1 M HCl. *Sci. Rep.* **13**, 942. 10.1038/s41598-023-27513-7 (2023). 36653379 10.1038/s41598-023-27513-7 PMC9849212

[56] Ebenso, E. E. et al. Molecular modelling of compounds used for corrosion Inhibition studies: a review. *Phys. Chem. Chem. Phys.* **23**, 19987 (2021). 34254097 10.1039/d1cp00244a

[57] Zhang, J. et al. *Corros. Sci.*, **53**, 1331–1336. (2011).

[58] Shin, M., Park, E. & Lee, H. *Plant-Inspired Pyrogallol-Containing Funct. Mater.*10.1002/adfm.201903022.

[59] Zheng, D., Wang, X., Zhang, M. & Ju, C. Synergistic effects between the two choline-based ionic liquids as lubricant additives in glycerol aqueous solution. *Tribol Lett.* **67**, 47 (2019).

[60] Ovri, J. E. O., Okeahialam, S. & Onyemaobi, O. Microbial corrosion of mild and medium carbon steels. *J. Eng. Sci. Technol. Rev.* **8**, 639–653 (2013).

[61] Li, Y. et al. Bacterial distribution in SRB biofilm affects mic pitting of carbon steel studied using FIB-SEM. *Corros. Sci.* 202

[62] Dadou, S. et al. The impact of halogen substitution on the corrosion Inhibition of Imidazothiazole derivatives for mild steel in corrosive media (Part A). *Colloids Surf., A*. **687**, 133451 (2024).

[63] Elyoussfi, A. et al. The effect of functional groups on the inhibitory efficacy of newly synthesized imidazopyridines compounds against the corrosion of mild steel in acidic environments: electrochemical, thermodynamic, surface and computational investigations (Part B). *J. Mol. Struct.* **1291**, 136025 (2023).

[64] Ebenso, E. E. et al. Molecular modelling of compounds used for corrosion Inhibition studies: a review. *Phys. Chem. Chem. Phys.* **23**(36), 19987–20027 (2021). 34254097 10.1039/d1cp00244a

[65] Wang, D. et al. Stress corrosion cracking behavior of X80 pipeline steel in acid soil environment with SRB. *Metall. Mater. Trans. A*. **48**, 2999–3007 (2017).

[66] Sun, D., Wu, M. & Xie, F. Effect of sulfate-reducing bacteria and cathodic potential on stress corrosion cracking of X70 steel in sea-mud simulated solution. *Mater. Sci. Eng. A*. **721**, 135–144 (2018).

[67] Bennet, D. O. Microbiology effective evaluation of biocide chemicals. In *SPE/IATMI Asia Pacific Oil & Gas Conference and Exhibition* (Society of Petroleum Engineers, 2017).

[68] Senthilmurugan, B. et al. Microbially induced corrosion in oilfield: microbial quantification and optimization of biocide application. *J. Chem. Technol. Biotechnol.* **94**, 2640–2650 (2019).

[69] Sahoo, J. et al. Synthesis, spectral characterization, in silico and in vitro antimicrobial investigations of some Schiff Base metal complexes derived fromazo salicylaldehyde analogues; NISCAIR-CSIR, (2016).

[70] Shaban, S. M. Studying the effect of newly synthesized cationic surfactant on silver nanoparticles formation and their biological activity. *J. Mol. Liq*. **216**, 137–145 (2016).

[71] Rbaa, M. et al. 8-Hydroxyquinoline based chitosan derived carbohydrate polymer as biodegradable and sustainable acid corrosion inhibitor for mild steel: experimental and computational analyses. *Int. J. Biol. Macromol.* **155**, 645–655 (2020). 32224172 10.1016/j.ijbiomac.2020.03.200

[72] Tonthat, N. K. et al. Structures of pathogenic fungal FKBP12s reveal possible self-catalysis function. *MBio* **7**, e00492–e00416 (2016). 27118592 10.1128/mBio.00492-16 PMC4850266

[73] Borisova, A. S. et al. Sequencing, biochemical characterization, crystal structure and molecular dynamics of cellobiohydrolase Cel7A from Geotrichum candidum 3C. *FEBS J.* **282**, 4515–4537 (2015). 26367132 10.1111/febs.13509

[74] Rodríguez-Cárdenas, Á. et al. Streptococcus pneumoniae TIGR4 flavodoxin: structural and biophysical characterization of a novel drug target. *PLoS ONE*. **11**, e0161020 (2016). 27649488 10.1371/journal.pone.0161020 PMC5029806

[75] Li, H. J. et al. Mechanism of the intramolecular Claisen condensation reaction catalyzed by MenB, a Crotonase superfamily member. *Biochemistry* **50**, 9532–9544 (2011). 21830810 10.1021/bi200877x PMC4119599

[76] Khowdiary, M. M., Taha, N. A., Saleh, N. M. & Elhenawy, A. A. Synthesis of novel nano-sulfonamide metal-based corrosion inhibitor surfactants. *Materials* **15**(3), 1146 (2022). 35161090 10.3390/ma15031146 PMC8838271

[77] Ahmed, A. et al. Molecular Docking and dynamics simulation revealed the potential inhibitory activity of aceis against SARS-CoV-2 targeting the hACE2 receptor. *Front. Chem.* **9**, 661230. 10.3389/fchem.2021.661230 (2021). 34017819 10.3389/fchem.2021.661230 PMC8129187

[78] Khelfaouia, H., Harkatia, D. & Salehb, B. A. Molecular docking, molecular dynamics simulations and reactivity, studies on approved drugs library targeting ACE2 and SARS-CoV-2 binding with ACE2. *J. Biomol. Struct. Dynamics*. **39**(18), 7246–7262. 10.1080/07391102.2020.1803967 (2021). 10.1080/07391102.2020.1803967 PMC7484571 32752951

